# Suicide and Cardiovascular Death Among Patients With Multiple Primary Cancers in the United States

**DOI:** 10.3389/fcvm.2022.857194

**Published:** 2022-06-06

**Authors:** Chen Su, Yan Wang, Fang Wu, Yumin Qiu, Jun Tao

**Affiliations:** ^1^Department of Hypertension and Vascular Disease, The First Affiliated Hospital, Sun Yat-sen University, Guangzhou, China; ^2^Key Laboratory on Assisted Circulation, Ministry of Health, Guangzhou, China; ^3^Department of Geriatrics, The First Affiliated Hospital, Sun Yat-sen University, Guangzhou, China

**Keywords:** cardiovascular diseases, suicide, multiple primary cancers, mortality risk, standardized mortality ratios

## Abstract

**Background:**

Previous studies have demonstrated that patients with a cancer diagnosis have an elevated risk of suicide and cardiovascular death. However, the effects of the diagnosis of multiple primary cancers (MPCs) on the risk of suicide and cardiovascular death remain unclear. This study aimed to identify the risk of suicide and cardiovascular death among patients with MPCs in the United States.

**Methods:**

Patients with a single or MPC(s) between 1975 and 2016 were selected from the Surveillance, Epidemiology, and End Results database in a retrospective cohort study. Mortality rates and standardized mortality ratios (SMRs) of suicides and cardiovascular diseases among patients with MPCs were estimated.

**Results:**

Of the 645,818 patients diagnosed with MPCs included in this analysis, 760 and 36,209 deaths from suicides and cardiovascular diseases were observed, respectively. The suicide and cardiovascular-disease mortality rates were 1.89- (95% CI, 1.76–2.02) and 1.65-times (95% CI, 1.63–1.67), respectively, that of the general population. The cumulative mortality rate from both suicides and cardiovascular diseases among patients with MPCs were significantly higher than those of patients with a single primary cancer (Both *p* < 0.001). In patients with MPCs diagnosed asynchronously, the cumulative incidence rates of suicides and cardiovascular deaths were higher than those diagnosed synchronously. Among all MPCs, cancers of the pancreas and esophagus had the highest SMRs of suicide (5.98 and 5.67, respectively), while acute myeloid leukemia and brain cancer had the highest SMRs of cardiovascular diseases (3.87 and 3.62, respectively). The SMR of suicide was highest within 1 year after diagnosis, while that of cardiovascular diseases was highest 5 years after diagnosis.

**Conclusions:**

This study showed that the mortality rates from suicides and cardiovascular diseases among patients with MPCs were higher than those with a single primary cancer. Therefore, our results underscore the need for psychological assessment and targeted preventive interventions for suicides and cardiovascular diseases among patients with MPCs.

## Introduction

As a consequence of improved cancer screening and treatments, there is a growing and aging population of cancer survivors. The amount of Americans living with cancer is predicted to reach 20.3 million people by 2026 (1, 2). However, compared to the general population, the elder age, genetic factors, and carcinogenic effects from anti-cancer treatment in cancer survivors will significantly contribute to the risks of developing new primary cancers (2, 3), which will, in turn, become the major life-threatening in cancer survivors. It is reported that MPCs accounts for 18.4% of all incident cancer diagnoses in the USA (4), and nearly 10% of cancer survivors are estimated to be with MPCs (5). Therefore, strengthening the comprehensive cognition of MPC patients' survivorship is an important part of optimizing the care of cancer patients and consolidating the medical effect. However, the researches on the description of survivorship of patients with MPCs remain limited.

Cancer patients had perpetually increased risks of suicide and serious cardiovascular death (CVD), either from shared lifestyles or from toxicities of cancer treatment (6–8). Previous studies have demonstrated that patients with a diagnosis of cancer are at high levels of distress and psychiatric symptoms (9–11). However, these studies are limited to the group of patients with single primary cancer (SPC). The effects of the diagnosis of MPCs on the risk of suicide and cardiovascular death remain largely unclear.

In this study, we conducted a population-based analysis to characterize the patterns and risk factors of deaths from suicides and cardiovascular events among patients with MPCs in the United States. Our findings will facilitate clinicians to screen patients with MPCs who are at high risk of suicide and cardiovascular death and provide personalized psychiatric care and better decision-making for cancer treatment.

## Methods

### Data Sources and Study Population

We used data from the Surveillance, Epidemiology, and End Results (SEER) program database to perform a retrospective cohort study. As a population-based cancer registry, the SEER database collects cancer data including demographics, incidence, anatomic site, morphology, stage, follow-up data, and treatment in the United States (12).

**Figure 1:**
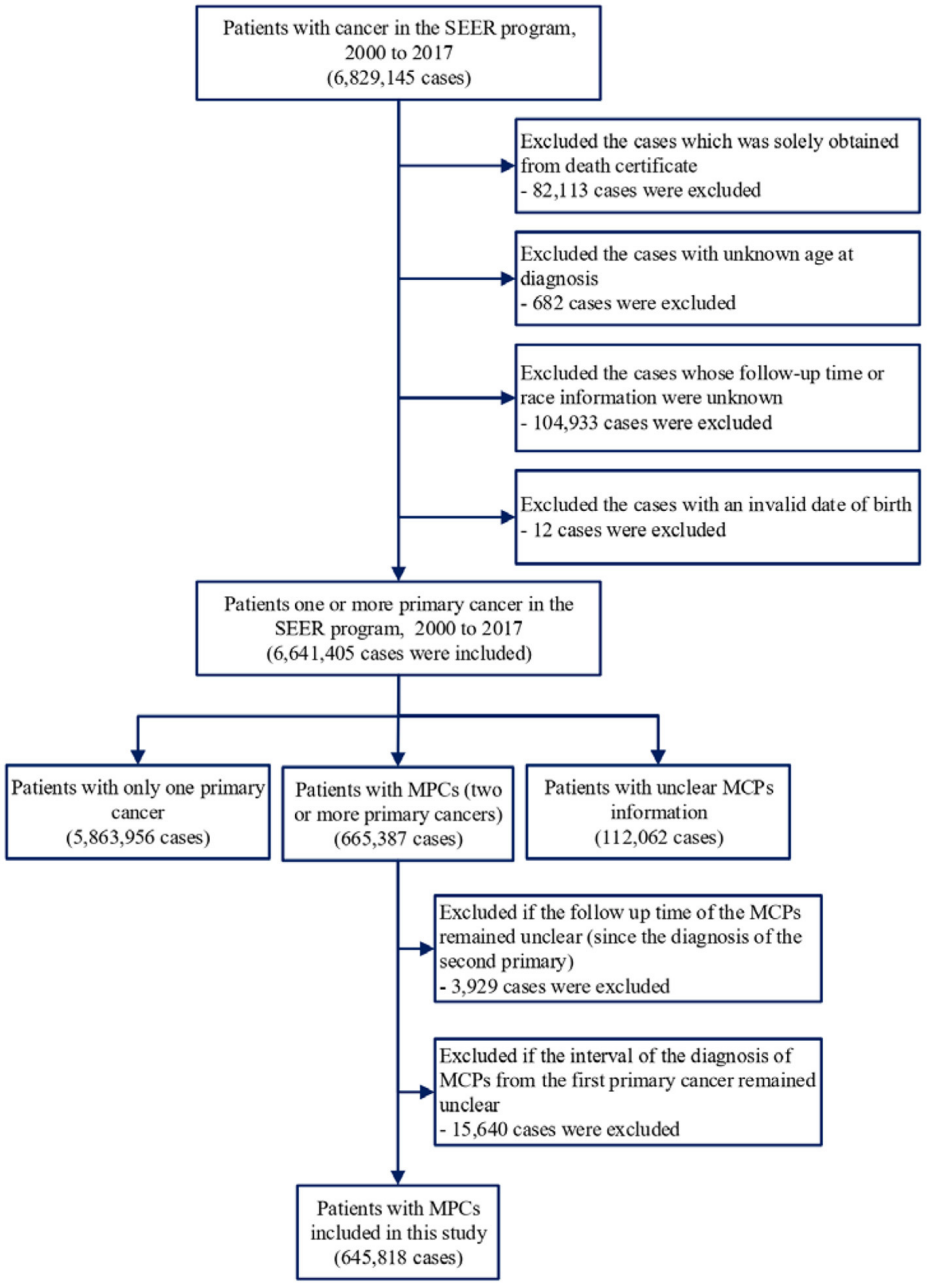
Inclusion and exclusion criteria for patients included in this study.

SEER^*^Stat software version 8.3.6 was applied to extract information on patients who were diagnosed with cancer between 1975 and 2016. We included patients with single or MPC(s) and excluded cancer patients diagnosed only by autopsy reports or death certificates. Patients without complete follow-up information, including age at diagnosis, follow-up duration, or race, were also excluded (Figure 1). For comparison with the general population, we obtained mortality data in the general US population registered by the National Center for Health Statistics between 1975 and 2016 (13).

### Study Variables

To simplify the analysis for MPCs, we diagnosed the second primary cancer as the start of follow-up. We investigated patients with MPCs, and for convenience, patients were followed up between the time of diagnosing the MPCs and death, exiting the study, or at the end of the study (December 31, 2016). The following variables were evaluated: age at diagnosis, sex, race, year of diagnosis, rural/urban status, median household income, cancer stage, surgical therapy, number of primary cancers, survival months, anatomic site, cause of death, and time from diagnosis. Notably, death from suicide or cardiovascular death was selected as the event of interest.

Patients with the cause of death coded as “50220” were considered to have died due to suicide, and “50060,” “50070,” “50080,” “50090,” “50100,” and “50110” were considered to have died due to cardiovascular death (14). The cause of death variable for suicide was 950-959 in the ICD-8 (International Statistical Classification of Diseases and Related Health Problems, eighth revision) codes for cases diagnosed between 1975 to 1978, 950-959 in the ICD-9 for cases diagnosed between 1979 and 1998, and U03, X60-X84, Y87.0 in the ICD-10 for cases diagnosed between 1999 and 2016. The cause of death variable for suicide was 390-448 in the ICD-8 codes for cases diagnosed between 1975 and 1978, 390-448 in the ICD-9 for cases diagnosed between 1979 and 1998, and I00-I78 in the ICD-10 for cases diagnosed between 1999 and 2016.

Additionally, because the shortest time interval was 1 month in the SEER database, patients whose survival duration was shorter than a month were recorded as 0 months. According to standard epidemiologic conventions, we changed records of “zero month” to “one-half month” (15).

### Statistical Analysis

The mortality rates from suicides or cardiovascular deaths among patients with MPCs were calculated as the number of deaths due to suicide or cardiovascular death divided by person-years of follow-up, while the standardized mortality ratios (SMRs) and corresponding 95% CIs in non-cancer deaths were calculated in accordance with previous studies (15–19). SMRs were defined as the ratio of the observed to the expected number of deaths. The expected values represented the number of deaths in the general population, which had a similar distribution of age, sex, calendar year, and race. For the standardization of age and calendar year, 5-year-categories were applied, and the values at the time of diagnosis were used. For standardization of race, white, black, and others were included. An approximation from the Poisson distribution was used to calculate the 95% CIs of the SMRs (15–17, 19). The cumulative incidence of suicide, cardiovascular diseases, heart diseases, and cerebrovascular diseases were calculated to show the difference between the number of primary cancers and the type of MPCs (20). Multivariable Cox analysis was used to compare the cumulative incidence of patients with MPCs to those with only one primary cancer. The SEER^*^Stat software, version 8.3.6 (US Department of Health and Human Services) and R version 3.52 (The R Project for Statistical Computing) statistical software package were used for analysis.

## Results

### Demographic Characteristics

Of the 645,818 cancer patients with MPCs included in the analysis (Figure 1), 760 deaths from suicides and 36,209 deaths from cardiovascular diseases were observed (Table 1). The suicide mortality rate was 32.8/100,000 person-years with an SMR of 1.89 (95% CI 1.76–2.02), while the cardiovascular mortality rate was 1,561.4/100,000 person-years with an SMR of 1.65 (95% CI 1.63–1.67). Cardiovascular deaths were mainly attributed to fatal heart diseases (77.7%) and cerebrovascular diseases (15.1%). Table 1 shows the baseline characteristics of the patients diagnosed with MPCs between 1975 and 2016. For patients with MPCs who committed suicide, a higher SMR was associated with age at diagnosis between 60 and 79 (2.10, 95% CI 1.92–2.29), male sex (1.94, 95% CI 1.80–2.10), black race (1.96, 95% CI 1.29–2.97), year of diagnosis between 2010 and 2018 (2.00, 95% CI 1.81–2.20), living in rural areas (2.53, 95% CI 2.12–3.02), low income (3.04, 95% CI 1.80–5.13), and distant stage of cancer (2.88, 95% CI 2.34–3.56). For patients with MPCs who died from cardiovascular diseases, a higher SMR was correlated with the American Indian or Alaska Native population (2.34, 95% CI 1.92–2.85), year of diagnosis between 2000 and 2004 (1.72, 95% CI 1.68–1.76), living in rural areas (1.85, 95% CI 1.79–1.90), low income (2.23, 95% CI 2.04–2.44), distant stage of disease (2.25, 95% CI 2.18–2.33), and receiving no surgery (2.05, 95% CI 2.02–2.08). Additionally, rates of death from cardiovascular diseases showed an increasing trend with age at diagnosis, and in those aged >80 years, the mortality rate was highest (SMR: 1.37, 95% CI 1.35–1.39).

**Table 1:** Suicide and cardiovascular death among patients with multiple primary cancers by baseline characteristics.

**Characteristics**	**No. of patients (%)**	**Follow up in total (person-years)**	**Suicides**			**Cardiovascular deaths**		
			**No. of observed deaths (%)**	**Mortality rate (per 100,000 person-years)**	**SMR (95% CI)^**a**^**	**No. of observed deaths (%)**	**Mortality rate (per 100,000 person-years)**	**SMR (95% CI)^**a**^**
All	645,818 (100%)	2,319,010	760 (100%)	32.8	1.89 (1.76–2.02)	36,209 (100%)	1,561.4	1.65 (1.63–1.67)
**Age**								
0–19	1,003 (0.2%)	4,250	0	0.0	0	5 (0.01%)	117.7	70.4 (29.3–169.0)
20–39	9,857 (1.5%)	52,626	12 (1.6%)	22.8	1.91 (1.09–3.37)	39 (0.1%)	74.1	5.34 (3.90–7.31)
40–59	118,701 (18.4%)	584,227	119 (15.7%)	20.4	1.32 (1.10–1.57)	1,976 (5.5%)	338.2	2.59 (2.48–2.70)
60–79	380,360 (58.9%)	1,369,337	486 (63.9%)	35.5	2.10 (1.92–2.29)	18,595 (51.4%)	1,358.0	1.90 (1.88–1.93)
80+	135,897 (21.0%)	308,570	143 (18.8%)	46.3	1.92 (1.63–2.26)	15,594 (43.1%)	5,053.6	1.37 (1.35–1.39)
**Sex**								
Female	297,060 (46.0%)	1,150,865	115 (15.1%)	10.0	1.63 (1.35–1.95)	13,714 (37.9%)	1,191.6	1.66 (1.63–1.69)
Male	348,758 (54.0%)	1,168,145	645 (84.9%)	55.2	1.94 (1.80–2.10)	22,495 (62.1%)	1,925.7	1.64 (1.62–1.67)
**Race**								
White	546,404 (84.6%)	1,990,451	723 (95.1%)	36.3	1.89 (1.76–2.04)	31,148 (86.0%)	1,564.9	1.62 (1.60–1.63)
Black	63,472 (9.8%)	200,987	22 (2.9%)	10.9	1.96 (1.29–2.97)	3,683 (10.2%)	1,832.5	1.83 (1.77–1.89)
AI/AN	2,517 (0.4%)	8,383	2 (0.3%)	23.9	3.31 (0.83–13.2)	99 (0.3%)	1,180.9	2.34 (1.92–2.85)
API	33,425 (5.2%)	119,188	13 (1.7%)	10.9	1.41 (0.82–2.43)	1,279 (3.5%)	1,073.1	2.15 (2.03–2.27)
**Year of diagnosis**								
2000–2004	68,510 (10.6%)	427,959	114 (15.0%)	26.6	1.67 (1.39–2.01)	7,542 (20.8%)	1,762.3	1.72 (1.68–1.76)
2005–2009	149,986 (23.2%)	799,884	242 (31.8%)	30.3	1.82 (1.61–2.07)	12,237 (33.8%)	1,529.8	1.66 (1.63–1.69)
2010–2018	427,322 (66.2%)	1,091,167	404 (53.2%)	37.0	2.00 (1.81–2.20)	16,430 (45.4%)	1,505.7	1.62 (1.59–1.64)
**Rural/urban status**								
Rural	78,055 (12.1%)	260,129	122 (16.1%)	46.9	2.53 (2.12–3.02)	4,662 (12.9%)	1,792.2	1.85 (1.79–1.90)
Urban	567,763 (87.9%)	2,058,881	638 (83.9%)	31.0	1.80 (1.66–1.94)	31,547 (87.1%)	1,532.2	1.63 (1.61–1.64)
**Median house-hold income**								
Low	9,329 (1.4%)	25,895	14 (1.8%)	54.1	3.04 (1.80–5.13)	472 (1.3%)	1,822.8	2.23 (2.04–2.44)
Median	431,774 (66.9%)	1,519,414	531 (69.9%)	34.9	2.01 (1.84–2.18)	25,044 (69.2%)	1,648.3	1.72 (1.70–1.74)
High	204,651 (31.7%)	773,360	215 (28.3%)	27.8	1.61 (1.41–1.84)	10,689 (29.5%)	1,382.2	1.49 (1.46–1.52)
Unknown	64 (0.01%)	341	0	0.0	0	4 (0.01%)	1,171.7	1.29 (0.48–3.44)
**Stage**								
In situ	4 (0.001%)	16	0	0.0	0	0	0.0	0
Localized	230,501 (35.7%)	1,360,673	331 (43.6%)	24.3	1.41 (1.27–1.57)	18,576 (51.3%)	1,365.2	1.48 (1.46–1.51)
Regional	86,089 (13.3%)	353,728	127 (16.7%)	35.9	2.35 (1.97–2.80)	5,380 (14.9%)	1,520.9	1.70 (1.66–1.75)
Distant	92,864 (14.4%)	172,702	87 (11.4%)	50.4	2.88 (2.34–3.56)	3,735 (10.3%)	2,162.7	2.25 (2.18–2.33)
Unstaged	236,360 (36.6%)	431,890	215 (28.3%)	49.8	2.54 (2.22–2.90)	8,518 (23.5%)	1,972.3	1.85 (1.81–1.89)
**Surgery**								
Yes	366,584 (56.8%)	1,704,318	438 (57.6%)	25.7	1.59 (1.45–1.75)	21,589 (59.6%)	1,266.7	1.46 (1.44–1.48)
No	272,970 (42.3%)	598,234	309 (40.7%)	51.7	2.47 (2.21–2.76)	14,323 (39.6%)	2,394.2	2.05 (2.02–2.08)
Unknown	6,264 (1.0%)	16,458	13 (1.7%)	79.0	4.14 (2.40–7.13)	297 (0.8%)	1,804.6	1.87 (1.67–2.09)

*SMR, standardized mortality ratios; CI, confidence interval; API, Asian or Pacific Islander; AI/AN, American Indian/Alaska Native*.

a *The SMRs were estimated as the ratios of observed to expected number of deaths. The observed values represented the number of deaths in cancer patients, whereas the expected values represented the number of individuals who died of the same causes in the general population, with a similar distribution of age, sex, race, and calendar year*.

### Comparison Between MPCs and SPC

**Figure 2:**
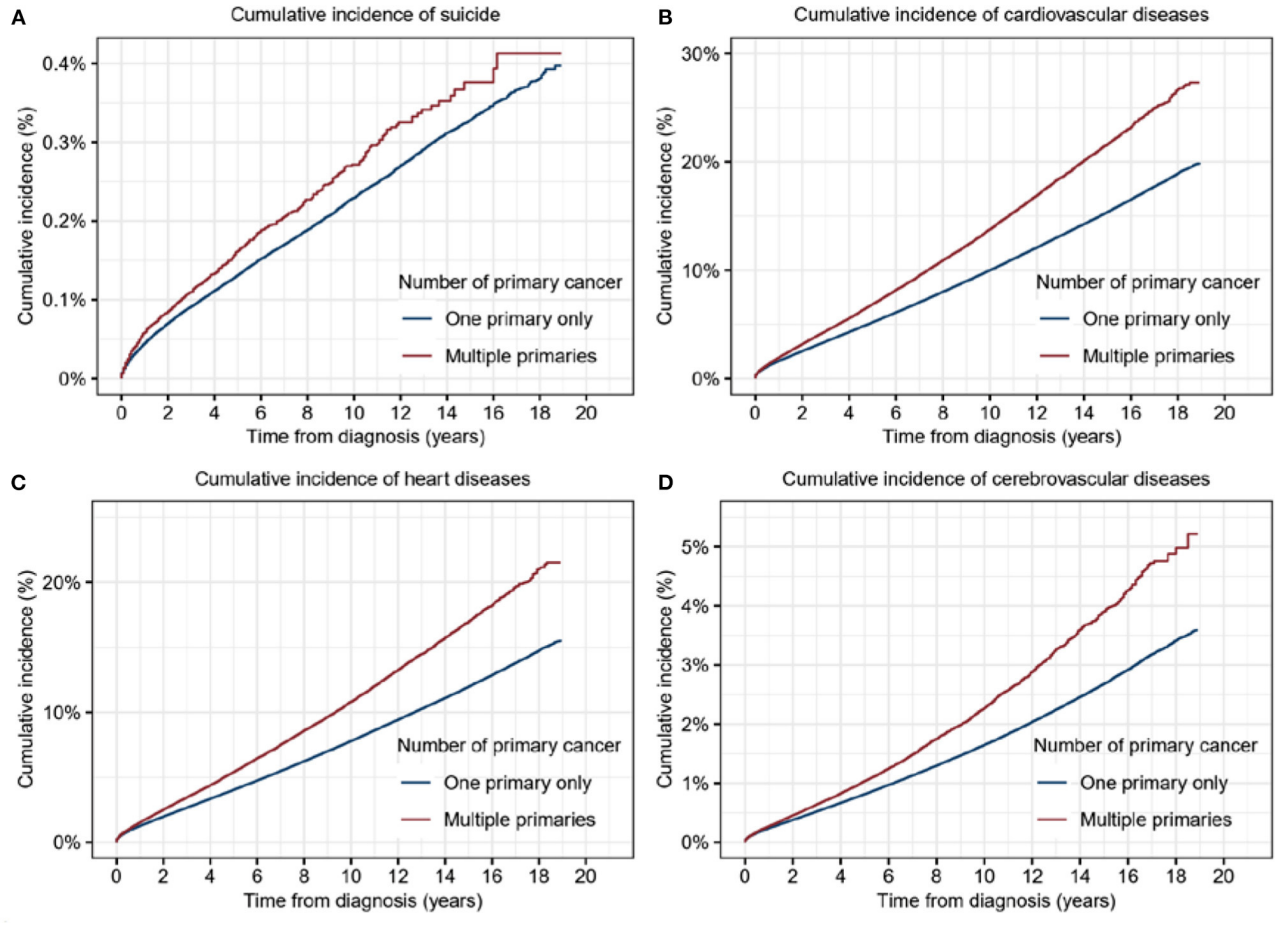
Cumulative incidence of suicide and cardiovascular diseases for patients with multiple primary cancers (MPCs) or single primary cancer (SPC). **(A)** Cumulative incidence of suicide for patients with MPCs or SPC. **(B)** Cumulative incidence of cardiovascular diseases for patients with MPCs or SPC. **(C)** Cumulative incidence of heart diseases for patients with MPCs or SPC. **(D)** Cumulative incidence of cerebrovascular diseases for patients with MPCs or SPC.

To reveal the differences in risk of suicide and CVD between patients with SPC and those with MPCs, we calculated the cumulative mortality of suicide and CVD for the distinct populations. The cumulative mortality of suicide in patients with MPCs was found significantly higher than that in those with an SPC (5-year cumulative mortality rate: 0.16% vs. 0.12%; *p* < 0.001) (Figure 2A). It remained true in the cumulative mortality of CVD (5-year cumulative mortality rate: 6.57% vs. 4.89%; *p* < 0.001) (Figure 2B), both in heart diseases (5-year cumulative mortality rate: 5.18% vs. 3.81%; *p* < 0.001) and cerebrovascular diseases (5-year cumulative mortality rate: 0.99% vs. 0.77%; *p* < 0.001) (Figures 2C,D). Univariable COX analysis demonstrated that patients with MPCs was found to had significantly higher risks of suicide (HR, 1.20; 95% CI, 1.12–1.3; *p* < 0.001) and cardiovascular deaths (HR, 1.35; 95% CI, 1.34–1.37; *p* < 0.001) than that in those with an SPC (Supplementary Table 1).

Multivariable Cox analysis further validated the above results (Table 2). Diagnosis with MPCs was proved as an independent risk factor for a higher risk of suicide without statistical significance (HR, 1.07; 95% CI, 1.00–1.16; *p* = 0.06). Male sex (HR, 4.77; 95% CI, 4.49–5.06; p < 0.001), older age at diagnosis (40–59: HR, 1.44, *p* < 0.001; 60–79: HR, 1.56, *p* < 0.001; 80+: HR, 2.00, *p* < 0.001), low income (HR, 1.34; 95% CI, 1.10–1.63; *p* < 0.001), distant stage (HR, 1.72; 95% CI, 1.60–1.85; *p* < 0.001), and not undergoing surgery (HR, 1.38; 95% CI, 1.31–1.45; *p* < 0.001) were positively related with suicide commitment.

**Table 2:** Multivariable Cox analysis to compare the cumulative mortality rate of suicide and cardiovascular deaths among patients with multiple primary cancers to those with only one primary cancer.

**Variables**	**Suicides**		**Cardiovascular deaths**	
	**HR (95% CI)**	**p**	**HR (95% CI)**	**p**
**Age at diagnosis**				
0–39	Reference		Reference	
40–59	1.44 (1.31–1.59)	<0.001	5.85 (5.57–6.15)	<0.001
60–79	1.56 (1.41–1.72)	<0.001	27.8 (26.5–29.2)	<0.001
80+	2.00 (1.78–2.24)	<0.001	128.6 (122.4–135.1)	<0.001
**Sex**				
Female	Reference		Reference	
Male	4.77 (4.49–5.06)	<0.001	1.25 (1.25–1.26)	<0.001
**Race**				
White	Reference		Reference	
Black	0.29 (0.26–0.33)	<0.001	1.33 (1.32–1.35)	<0.001
API	0.60 (0.53–0.67)	<0.001	0.79 (0.77–0.8)	<0.001
AI/AN	1.10 (0.81–1.49)	0.5	0.99 (0.94–1.05)	0.8
**Year of diagnosis**				
2000–2004	Reference		Reference	
2005–2009	0.98 (0.92–1.03)	0.4	0.81 (0.8–0.82)	<0.001
2010–2018	0.96 (0.91–1.02)	0.2	0.66 (0.65–0.67)	<0.001
**Median house-hold income**				
Low	1.34 (1.10–1.63)	0.003	1.43 (1.39–1.48)	<0.001
Median	1.27 (1.21–1.33)	<0.001	1.18 (1.17–1.19)	<0.001
High	Reference		Reference	
**Rural/urban status**				
Rural	Reference		Reference	
Urban	0.85 (0.79–0.90)	<0.001	0.93 (0.92–0.94)	<0.001
**Stage**				
In situ	1.13 (1.02–1.26)	0.02	0.93 (0.91–0.94)	<0.001
Localized	Reference		Reference	
Regional	1.71 (1.61–1.83)	<0.001	1.34 (1.32–1.35)	<0.001
Distant	1.72 (1.60–1.85)	<0.001	1.42 (1.41–1.44)	<0.001
Unstaged	1.39 (1.31–1.48)	<0.001	1.36 (1.35–1.37)	<0.001
**Surgery**				
Yes	Reference		Reference	
No	1.38 (1.31–1.45)	<0.001	1.39 (1.38–1.4)	<0.001
**Number of primary cancers**				
Only one	Reference		Reference	
MPCs	1.07 (1.00–1.16)	0.06	1.02 (1.01–1.03)	0.004

*HR, Hazard ratios; API, Asian or Pacific Islander; AI/AN, American Indian/Alaska Native; MPCs, multiple primary cancers*.

Moreover, the risk of cardiovascular death was also observed to be significantly higher in patients with MPCs, compared with those with SPC in the multivariable analyses (HR, 1.02; 95% CI, 1.01–1.03; *p* = 0.004). Other risk factors of cardiovascular death included older age (40–59: HR, 5.85, *p* < 0.001; 60–79: HR, 27.8, *p* < 0.001; 80+: HR, 128.6, *p* < 0.001), male sex (HR, 1.25; 95% CI, 1.25–1.26; p < 0.001), black race (HR, 1.33; 95% CI, 1.32–1.35; *p* < 0.001), low income (HR, 1.43; 95% CI, 1.39–1.48; *p* < 0.001), distant stage of cancer (HR, 1.42; 95% CI, 1.41–1.44; *p* < 0.001), and receiving no surgery (HR, 1.50; 95% CI, 1.49–1.51, *p* < 0.001).

We further performed multivariable Cox analysis adjusted by baseline characteristics of age, sex, race, and year of diagnosis (Supplementary Table 2). The results showed that there was no significant difference in the risk of suicide (HR, 1.07; 95% CI, 0.99–1.16; *p* = 0.07) and cardiovascular deaths (HR, 1.00; 95% CI, 0.99–1.01; *p* = 0.5) between patients with MPCs and SPC, indicating the underlying impacts from confounding factors.

### Comparison of Synchronous and Asynchronous MPCs

**Figure 3:**
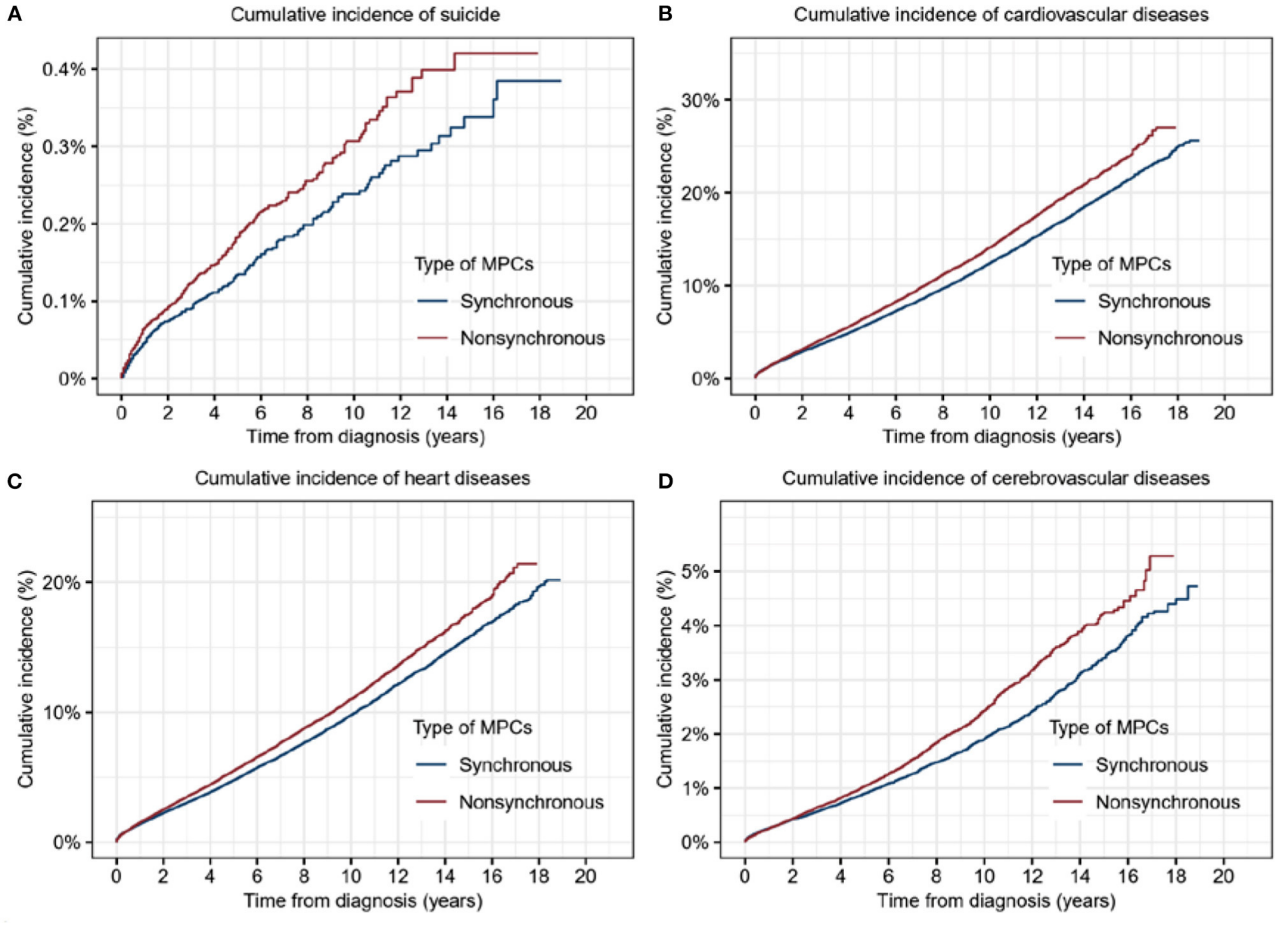
Cumulative incidence of suicide and cardiovascular diseases for patients with synchronous multiple primary cancers (MPCs) or asynchronous MPCs. **(A)** Cumulative incidence of suicide for patients with synchronous MPCs or asynchronous MPCs. **(B)** Cumulative incidence of cardiovascular diseases for patients with synchronous MPCs or asynchronous MPCs. **(C)** Cumulative incidence of heart diseases for patients with synchronous MPCs or asynchronous MPCs. **(D)** Cumulative incidence of cerebrovascular diseases for patients with synchronous MPCs or asynchronous MPCs.

According to the interval from the first primary cancer, the MPCs were classified as synchronous or asynchronous subtypes. Synchronous cancers refer to MPCs diagnosed within 1 year following the first primary cancer, while those beyond 1 year from the first primary belong to the asynchronous group. To be more precise, asynchronous MPC with a diagnosis interval <5 years from the first primary tumor was included in this analysis. Higher cumulative incidence of both suicide (*p* = 0.003) and CVD (*p* < 0.001), including heart diseases (*p* < 0.001) and cerebrovascular diseases (*p* < 0.001), were significantly associated with synchronous MPCs (Figure 3).

### Suicide and Cardiovascular Death of Patients With MPCs by Cancer Type

In the subgroup analyses stratified by cancer types, the majority of deaths from suicide occurred in patients with MPCs of the lung and bronchus (14.2%), prostate (13.0%), colon and rectum (12.1%), urinary bladder (7.9%), and non-basal skin (7.6%), which accounted for more than half of the total suicidal deaths. Among all types of MPCs, the SMR for suicide was observed greatest in the cancers of the pancreas (5. 98, 95% CI 3.81–9.37), followed by cancers of the esophagus (5.67, 95% CI 3.42–9.41), acute myeloid leukemia (4.72, 95% CI 2.46–9.07), larynx (4.16, 95% CI 2.58–6.68), and oral cavity and pharynx (3.68, 95% CI 2.76–4.92) (Table 3).

**Table 3:** Suicide and cardiovascular deaths among patients with multiple primary cancers by cancer types.

**Cancer types**	**No. of patients (%)**	**Follow up in total (person-years)**	**Causes of death**					
			**Suicides**			**Cardiovascular deaths**		
			**No. of observed deaths (%)**	**Mortality rate (per 100,000 person-years)**	**SMR (95% CI)^**a**^**	**No. of observed deaths (%)**	**Mortality rate (per 100,000 person-years)**	**SMR (95% CI)^**a**^**
All	645,818 (100%)	2,319,010	760 (100%)	32.8	**1.89 (1.76–2.02)**	36,209 (100%)	1,561.40	**1.65 (1.63–1.67)**
Pancreas	16,815 (2.6%)	16,926	19 (2.5%)	112.3	**5.98 (3.81–9.37)**	380 (1.0%)	2,245.10	**2.26 (2.04–2.50)**
Esophagus	6,960 (1.1%)	10,934	15 (2.0%)	137.2	**5.67 (3.42–9.41)**	263 (0.7%)	2,405.40	**2.14 (1.90–2.42)**
Acute myeloid leukemia	9,997 (1.5%)	11,066	9 (1.2%)	81.3	**4.72 (2.46–9.07)**	256 (0.7%)	2,313.40	**3.87 (3.42–4.37)**
Larynx	5,094 (0.8%)	17,287	17 (2.2%)	98.3	**4.16 (2.58–6.68)**	388 (1.1%)	2,244.40	**2.09 (1.89–2.31)**
Oral cavity and pharynx	17,825 (2.8%)	57,877	46 (6.1%)	79.5	**3.68 (2.76–4.92)**	939 (2.6%)	1,622.40	**1.90 (1.78–2.02)**
Small intestine	4,464 (0.7%)	15,016	9 (1.2%)	59.9	**3.41 (1.78–6.56)**	255 (0.7%)	1,698.20	**1.88 (1.66–2.13)**
Lung and bronchus	97,828 (15.1%)	197,676	108 (14.2%)	54.6	**3.28 (2.72–3.96)**	4,220 (11.7%)	2,134.80	**2.17 (2.11–2.24)**
Anus	2,753 (0.4%)	10,059	5 (0.7%)	49.7	**3.15 (1.31–7.58)**	125 (0.3%)	1,242.60	**1.78 (1.49–2.12)**
Stomach	10,446 (1.6%)	21,774	11 (1.4%)	50.5	**2.73 (1.51–4.94)**	551 (1.5%)	2,530.50	**2.27 (2.08–2.46)**
Corpus uteri	14,666 (2.3%)	67,960	10 (1.3%)	14.7	**2.23 (1.20–4.15)**	526 (1.5%)	774	**1.53 (1.40–1.66)**
Colon and rectum	64,427 (10.0%)	249,792	92 (12.1%)	36.8	**2.05 (1.67–2.52)**	5,106 (14.1%)	2,044.10	**1.66 (1.61–1.70)**
Endocrine system	14,483 (2.2%)	74,016	19 (2.5%)	25.7	**1.84 (1.17–2.89)**	429 (1.2%)	579.6	**1.36 (1.24–1.50)**
Non-Hodgkin lymphoma	29,576 (4.6%)	106,094	33 (4.3%)	31.1	**1.63 (1.16–2.30)**	1,693 (4.7%)	1,595.80	**1.57 (1.50–1.65)**
Prostate	50,554 (7.8%)	251,616	99 (13.0%)	39.3	**1.43 (1.17–1.74)**	3,507 (9.7%)	1,393.80	**1.28 (1.24–1.32)**
Urinary bladder	41,345 (6.4%)	166,221	60 (7.9%)	36.1	**1.40 (1.09–1.81)**	3,744 (10.3%)	2,252.40	**1.55 (1.50–1.60)**
Vulva	2,397 (0.4%)	9,427	2 (0.3%)	21.2	3.37 (0.84–13.5)	151 (0.4%)	1,601.70	**1.74 (1.49–2.04)**
Chronic myeloid leukemia	2,568 (0.4%)	7,804	4 (0.5%)	51.3	2.61 (0.98–6.96)	186 (0.5%)	2,383.40	**2.52 (2.18–2.91)**
Ureter	1,515 (0.2%)	5,239	3 (0.4%)	57.3	2.51 (0.81–7.80)	103 (0.3%)	1,965.90	**1.54 (1.27–1.86)**
Ovary	7,376 (1.1%)	30,265	4 (0.5%)	13.2	1.89 (0.71–5.04)	193 (0.5%)	637.7	**1.65 (1.43–1.90)**
Soft tissue including heart	4,647 (0.7%)	15,345	5 (0.7%)	32.6	1.72 (0.71–4.13)	199 (0.5%)	1,296.90	**1.38 (1.21–1.59)**
Hodgkin lymphoma	1,598 (0.2%)	6,612	2 (0.3%)	30.2	1.62 (0.40–6.46)	78 (0.2%)	1,179.60	**1.97 (1.58–2.46)**
Brain	5,290 (0.8%)	7,414	2 (0.3%)	27	1.51 (0.38–6.03)	130 (0.4%)	1,753.50	**3.62 (3.05–4.30)**
Kidney and renal pelvis	30,032 (4.7%)	129,095	33 (4.3%)	25.6	1.28 (0.91–1.80)	1,818 (5.0%)	1,408.30	**1.70 (1.62–1.78)**
Non-basal skin	42,786 (6.6%)	199,563	58 (7.6%)	29.1	1.28 (0.99–1.65)	2,756 (7.6%)	1,381.00	**1.33 (1.28–1.38)**
Testis	1,044 (0.2%)	6,333	2 (0.3%)	31.6	1.22 (0.31–4.89)	15 (0.04%)	236.9	1.33 (0.80–2.21)
Breast	90,541 (14.0%)	455,443	33 (4.3%)	7.2	1.12 (0.79–1.57)	4,104 (11.3%)	901.1	**1.41 (1.37–1.45)**
Liver	9,103 (1.4%)	13,949	3 (0.4%)	21.5	1.08 (0.35–3.34)	278 (0.8%)	1,993.00	**2.38 (2.11–2.67)**
Myeloma	9,188 (1.4%)	26,488	5 (0.7%)	18.9	0.99 (0.41–2.38)	621 (1.7%)	2,344.40	**2.18 (2.01–2.36)**
Chronic lymphocytic leukemia	6,879 (1.1%)	28,235	5 (0.7%)	17.7	0.81 (0.34–1.94)	527 (1.5%)	1,866.50	**1.44 (1.32–1.57)**
Cervix uteri	2,132 (0.3%)	8,122	0 (0.0%)	0	0	78 (0.2%)	960.3	**2.28 (1.83–2.85)**

*SMR, standardized mortality ratios; CI, confidence interval*.

a *The SMRs were estimated as the ratios of observed to expected number of deaths. The observed values represented the number of deaths in cancer patients, whereas the expected values represented the number of individuals who died of the same causes in the general population, with a similar distribution of age, sex, race, and calendar year. The bold values mean p-value < 0.05*.

In the terms of CVD mortality risk (Table 3), the plurality of CVD death occurred in patients with cancers of the colon and rectum (14.1%), lung and bronchus (11.7%), breast (11.3%), urinary bladder (10.3%), and prostate (9.7%). Certain MPC patients have relatively high SMR from CVD. For instance, patients with acute myeloid leukemia had an SMR of 3.87 (95% CI 3.42–4.37), and those with brain cancer had an SMR of 3.62 (95% CI 3.05–4.30).

### Suicide and Cardiovascular Death of Patients With MPCs by Combination of First Primary Cancer and Second Primary Cancer

We further explored which combination of first primary cancer and second primary cancer was related with the highest risk of suicide and cardiovascular death (Supplementary Tables S3, S4). Combination of oral cavity and pharynx cancer and lung cancer had highest risk of suicide (SMR: 9.40; 95% CI, 5.76–15.3), followed by combination of prostate cancer and pancreas cancer (SMR: 9.30; 95% CI, 5.15–16.8), combination of two different oral cavity and pharynx cancer (SMR: 5.64; 95% CI, 3.64–8.74), and combination of prostate cancer and esophagus cancer (SMR: 5.26; 95% CI, 2.19–12.6). Combination of breast cancer and acute lymphocytic leukemia had highest risk of cardiovascular deaths (SMR: 9.40; 95% CI, 5.76–15.3), combination of two different brain cancer (SMR: 8.48; 95% CI, 4.24–17.0), and combination of oral cavity and pharynx cancer and acute myeloid leukemia (SMR: 8.05; 95% CI, 3.35–19.3). More cancer combinations can be found in Supplementary Tables S1, S2.

### Suicide and Cardiovascular Death by Time After MPCs Diagnosis

Overall, the SMR of suicide and CVD among MPCs patients remained significantly elevated throughout all follow-up times despite fluctuations. Compared with the general population, the risk of suicide was immediately elevated within 1 year following diagnosis (SMR, 3.48, 95% CI 3.11–3.89), which gradually subsided with a longer follow-up time. Similarly, the SMR of CVD prominently declined during 1–5 years after diagnosis, compared to that within the first year following diagnosis. However, this SMR peaked at over 5 years after diagnosis (Table 4).

**Table 4:** Suicide and cardiovascular death among patients with multiple primary cancers by time from diagnosis.

**Time from diagnosis**	**No. of patients (%)**	**Follow up in total (person-years)**	**Suicides**			**Cardiovascular deaths**		
			**No. of observed deaths (%)**	**Mortality rate (per 100,000 person-years)**	**SMR (95% CI)^**a**^**	**No. of observed deaths (%)**	**Mortality rate (per 100,000 person-years)**	**SMR (95% CI) ^**a**^**
All	645,818	2,319,010	760 (100.0%)	32.8	1.89 (1.76–2.02)	36,209 (100.0%)	1,561.4	1.65 (1.63–1.67)
Within 1 year	645,818	474,696	309 (40.7%)	65.1	3.48 (3.11–3.89)	10,414 (28.8%)	2,193.8	1.91 (1.87–1.94)
1–5 years	434,348	1,137,197	295 (38.8%)	25.9	1.46 (1.31–1.64)	14,528 (40.1%)	1,277.5	1.29 (1.27–1.32)
5+ years	178,317	707,118	156 (20.5%)	22.1	1.38 (1.18–1.62)	11,267 (31.1%)	1,593.4	2.14 (2.11–2.18)

*SMR, standardized mortality ratios; CI, confidence interval; API, Asian or Pacific Islander; AI/AN, American Indian/Alaska Native*.

^a^*The SMRs were estimated as the ratios of observed to expected number of deaths. The observed values represented the number of deaths in cancer patients, whereas the expected values represented the number of individuals who died of the same causes in the general population, with a similar distribution of age, sex, race, and calendar year*.

## Discussion

We present a contemporary population-based analysis of the risk of suicide and CVD among over 640,000 MPCs patients and revealed that risks of suicide and CVD death varied as a function of age, gender, race, cancer type, time since initial cancer diagnosis, and follow-up time. The mortality rates from suicide and CVD in MPCs patients were significantly higher than those with SPC. Diagnosis with MPCs represents a great challenge not only in cancer management but also in the psychological construction of patients. The participants in healthcare should be aware of this underlying risk and implement timely targeted interventions for subpopulations at elevated risk.

The relative risk of suicide among cancer patients was nearly 2-folds that of the general population, and MPCs patients exhibited apparently higher cumulative mortality of suicide than those with SPC. The results of the current work suggested that suicide-prevention strategies should be aimed at patients with the MPCs of the pancreas, esophagus, and larynx, as well as acute myeloid leukemia. Our finding was supported by the work by Henson et al., who reported that patients with primary cancer of the pancreas and esophagus have the highest risk of suicide in England (21). This was associated with the psychological stress caused by the negative prognosis, which could be attributed to limited treatment options and severely impaired quality of life (22, 23). Poor physical health was also a driver of suicide, even after adjusting for depression and other covariates (24). Patients with esophageal and laryngeal cancers have a strong association with alcohol consumption and tobacco use, which are demonstrated risk factors contributing to suicide in the general population (25, 26).

MPCs survivors, when compared to single cancer survivors, had a lower global quality of life, poorer emotional role function, greater and more frequent distress, and greater sub-clinical anxiety (3, 27), all of which would facilitate the suicide ideation and commitment. Additionally, we found SMR of suicide was highest within the first year after diagnosis during the follow-up period, consistent with previous studies (22, 28). This may be ascribed to the immediate blow of the diagnosis to patients' psychology.

We observed that MPCs patients are perpetually at elevated risk of death from CVD compared to the general population, as well as comparison with SPC patients. Consistent with previous reports, younger age of diagnosis is associated with higher SMR (29), and the first year of cancer diagnosis represents the period with extended risks for both SMR and mortality rate of CVD (30, 31). These findings might also be partially explained by the negative psychological stress, as well as the aggressive treatment shortly after cancer discovery and long-time at risk until the death of the general population (32, 33). It could also be modulated from the fact that patients who die early are those with severe co-existing CVDs (34). Patients with co-existing CVDs at cancer diagnosis are also at high risk of cardiotoxicity from cancer treatment (35, 36). Additionally, MPCs patients are reported more likely to indulge in alcohol and smoke than those with SPC (37). Although this study could not fully confirm the causal relationship between smoking and drinking and MPC, perhaps they are reciprocal causation, tobacco and alcohol have been proved considerable risk factors for serious CVD. Consistently, a cohort study reported that patients with MPCs have a greater risk of developing stroke, coronary artery disease, arrhythmia, pericarditis, and valvular heart disease (37).

When stratified by cancer site, the relative risk of CVD was found higher in MPCs patients with acute myeloid leukemia, brain cancer, and chronic myeloid leukemia. Several pathways for increased risk of stroke in cancer patients have been proposed, and there are several cancer-specific types and causes of stroke in cancer patients (38, 39). First, certain cancers cause occlusive disease from emboli, compression, or meningeal extension of tumor. Patients with leukemia and elevated leukocyte counts may develop intravascular leukostasis, leading to hemorrhagic infarct, and brain tumor metastases may also cause hemorrhage. Second, coagulopathies, including non-bacterial thrombotic endocarditis, could cause serious CVD. Brain tumor metastases may cause venous thromboembolism (40), and studies have shown that brain cancer patients have a dramatically elevated SMR from stroke of 7.63 in the first 5 years after diagnosis (41). Third, CVD may occur from cancer treatment. Tyrosine kinase inhibitors (TKIs) are widely used for the treatment of chronic myeloid leukemia. Studies have found that TKIs can increase the risk of acute coronary syndromes, heart failure, arrhythmias, and venous thrombosis (42, 43). Cyclophosphamide, commonly used in the treatment of leukemia, is known to have considerable cardiotoxicity. These studies greatly provided evidence and explanations for our results.

### Limitations

This study has several limitations. First, the risk of bias on reporting death certificates may exist, which may result in incorrect classification of the causes of death (44, 45). However, the National Vital Statistics System and National Center for Health Statistics are responsible for collecting the SEER mortality data, which ensures that the data collection procedures are standardized and accurate (46). Previous studies also confirmed that death certificates in SEER are acceptable and have high validity and reliability (47). Secondly, the SEER database does not provide information on comorbidities, psychiatric conditions, quality of life, social support, tobacco consumption, dependence on alcohol, and previous cardiovascular diseases. Thus, it is difficult to perform further analysis on the association between these factors and the risk of death from suicide and cardiovascular death. Nonetheless, the SEER database collects an extensive amount of data, and is still a useful and powerful tool in clinical research and exploration (45). Finally, we only compared the mortality rate of patients with second primary cancer and those with single primary cancer. It is more complicated when there are three or more cancers in the same patients; thus, we did not analyze this proportion of patients independently in the study. Further studies are warranted to address this problem.

## Conclusion

This study demonstrated, for the first time, that the mortality rates from suicide and cardiovascular diseases among patients with MPCs were significantly higher than those of patients with SPC in the United States. The mortality rates from suicide and cardiovascular diseases among patients with MPCs were 1.89 times and 1.65 times those of the general population, respectively. In addition, patients with asynchronous MPCs had a higher risk of suicide and cardiovascular death than those with synchronous MPCs. The highest SMR of suicide was observed in MPCs of the pancreas and was within the first year after diagnosis of the MPCs, while the highest SMR of cardiovascular death was found in MPCs of acute myeloid leukemia and after 5 years after diagnosis of the MPCs. Diagnosis with MPCs represents a great challenge not only in cancer management but also in the psychological construction of patients. The participants in healthcare should be aware of this underlying risk and implement timely targeted interventions for subpopulations at elevated risk.

## Data Availability Statement

Publicly available datasets were analyzed in this study. This data can be found here: https://seer.cancer.gov/.

## Ethics Statement

This study was reviewed and approved by the ethics committee of The First Affiliated Hospital, Sun Yat-sen University.

## Author Contributions

JT had full access to all of the data in the study and takes responsibility for the integrity of the data and the accuracy of the data analysis, concept and design, and funding. CS, YW, FW, and JT: acquisition, analysis, or interpretation of data. CS, YW, YQ, and JT: drafting of the manuscript. CS and JT: statistical analysis and supervision. CS, FW, and JT: administrative, technical, or material support. All authors: critical revision of the manuscript for important intellectual content. All authors contributed to the article and approved the submitted version.

## Funding

This work was supported by the National Nature Science Foundation of China (82000461) and Medical Scientific Research Foundation of Guangdong Province of China (C201903).

## Conflict of Interest

The authors declare that the research was conducted in the absence of any commercial or financial relationships that could be construed as a potential conflict of interest.

## Publisher's Note

All claims expressed in this article are solely those of the authors and do not necessarily represent those of their affiliated organizations, or those of the publisher, the editors and the reviewers. Any product that may be evaluated in this article, or claim that may be made by its manufacturer, is not guaranteed or endorsed by the publisher.
