# Supplementary material for: Suicide and Cardiovascular Death Among Patients With Multiple Primary Cancers in the United States
Source: Front Cardiovasc Med. 2022 Jun 6;9:857194. doi: 10.3389/fcvm.2022.857194 (PMC9208264; doi:10.3389/fcvm.2022.857194)
Supplement: Supplementary file 1 [file Data_Sheet_1.DOCX]

**Supplementary Table Legend**

**Table S1** Univariable Cox analysis to compare the cumulative mortality rate of suicide and cardiovascular deaths among patients with multiple primary cancers to those with only one primary cancer.

**Table S2** Multivariable Cox analysis by age, sex, race and year to compare the cumulative mortality rate of suicide and cardiovascular deaths among patients with multiple primary cancers to those with only one primary cancer.

**Table S3** Suicides among patients with multiple primary cancers by combination of first primary cancer and second primary cancer.

**Table S4** Cardiovascular deaths among patients with multiple primary cancers by combination of first primary cancer and second primary cancer.

**Notes:** For **Table S3** and **Table S4**, only the combinations of first primary cancer and second primary cancer with a target event (Suicides or cardiovascular deaths) of at least 5 deaths were included, as there were over 2,000 types of combinations of first primary cancer and second primary cancer and most of they had a target event of less than five cases.

**Table S1** Univariable Cox analysis to compare the cumulative mortality rate of suicide and cardiovascular deaths among patients with multiple primary cancers to those with only one primary cancer.

| Variables | Suicides | |  | Cardiovascular deaths | |
| --- | --- | --- | --- | --- | --- |
|  | HR (95% CI) | p |  | HR (95% CI) | p |
| Age at diagnosis |  |  |  |  |  |
| 0-39 | Reference |  |  | Reference |  |
| 40-59 | 1.34 (1.21-1.48) | < 0.001 |  | 5.39 (5.13-5.67) | < 0.001 |
| 60-79 | 1.81 (1.65-1.99) | < 0.001 |  | 27.2 (25.9-28.5) | < 0.001 |
| 80+ | 2.19 (1.96-2.45) | < 0.001 |  | 125.1 (119.1-131.4) | < 0.001 |
| Sex |  |  |  |  |  |
| Female | Reference |  |  | Reference |  |
| Male | 4.99 (4.71-5.28) | < 0.001 |  | 1.47 (1.46-1.48) | < 0.001 |
| Race |  |  |  |  |  |
| White | Reference |  |  | Reference |  |
| Black | 0.31 (0.28-0.35) | < 0.001 |  | 1.16 (1.14-1.17) | < 0.001 |
| API | 0.52 (0.47-0.58) | < 0.001 |  | 0.69 (0.68-0.7) | < 0.001 |
| AI/AN | 1.11 (0.83-1.5) | 0.5 |  | 0.75 (0.71-0.8) | 0.8 |
| Year of diagnosis |  |  |  |  |  |
| 2000-2004 | Reference |  |  | Reference |  |
| 2005-2009 | 1 (0.95-1.06) | 1 |  | 0.82 (0.81-0.82) | < 0.001 |
| 2010-2018 | 1 (0.94-1.06) | 1 |  | 0.68 (0.68-0.69) | < 0.001 |
| Median house-hold income |  |  |  |  |  |
| Low | 1.61 (1.34-1.93) | 0.003 |  | 1.49 (1.45-1.53) | < 0.001 |
| Median | 1.3 (1.24-1.37) | < 0.001 |  | 1.22 (1.21-1.23) | < 0.001 |
| High | Reference |  |  | Reference |  |
| Rural/urban status |  |  |  |  |  |
| Rural | Reference |  |  | Reference |  |
| Urban | 0.71 (0.67-0.76) | < 0.001 |  | 0.8 (0.8-0.81) | < 0.001 |
| Stage |  |  |  |  |  |
| In situ | 0.66(0.6-0.74) | 0.02 |  | 0.64(0.63-0.65) | < 0.001 |
| Localized | Reference |  |  | Reference |  |
| Regional | 1.19 (1.12-1.27) | < 0.001 |  | 1.04 (1.03-1.05) | < 0.001 |
| Distant | 1.78 (1.66-1.9) | < 0.001 |  | 1.46 (1.44-1.48) | < 0.001 |
| Unstaged | 1.41 (1.33-1.5) | < 0.001 |  | 1.32 (1.31-1.34) | < 0.001 |
| Surgery |  |  |  |  |  |
| Yes | Reference |  |  | Reference |  |
| No | 2.12 (2.03-2.22) | < 0.001 |  | 2.11 (2.1-2.13) | < 0.001 |
| Number of primary cancers |  |  |  |  |  |
| Only one | Reference |  |  | Reference |  |
| MPCs | 1.2 (1.12-1.3) | < 0.001 |  | 1.35 (1.34-1.37) | < 0.001 |

Abbreviations: HR: Hazard ratios; API: Asian or Pacific Islander; AI/AN: American Indian/Alaska Native; MPCs: multiple primary cancers.

**Table S2** Multivariable Cox analysis by age, sex, race and year to compare the cumulative mortality rate of suicide and cardiovascular deaths among patients with multiple primary cancers to those with only one primary cancer.

| Variables | Suicides | |  | Cardiovascular deaths | |
| --- | --- | --- | --- | --- | --- |
|  | HR (95% CI) | p |  | HR (95% CI) | p |
| Age at diagnosis |  |  |  |  |  |
| 0-39 | Reference |  |  | Reference |  |
| 40-59 | 1.32 (1.2-1.46) | < 0.001 |  | 5.36 (5.10-5.64) | < 0.001 |
| 60-79 | 1.45 (1.32-1.59) | < 0.001 |  | 26.2 (24.9-27.5) | < 0.001 |
| 80+ | 1.93 (1.73-2.16) | < 0.001 |  | 125.1 (119.1-131.3) | < 0.001 |
| Sex |  |  |  |  |  |
| Female | Reference |  |  | Reference |  |
| Male | 4.91 (4.64-5.2) | < 0.001 |  | 1.34 (1.33-1.35) | < 0.001 |
| Race |  |  |  |  |  |
| White | Reference |  |  | Reference |  |
| Black | 0.31 (0.27-0.35) | < 0.001 |  | 1.41 (1.40-1.43) | < 0.001 |
| API | 0.59 (0.52-0.66) | < 0.001 |  | 0.77 (0.76-0.78) | < 0.001 |
| AI/AN | 1.22 (0.9-1.65) | 0.2 |  | 1.06 (1.00-1.12) | 0.05 |
| Year of diagnosis |  |  |  |  |  |
| 2000-2004 | Reference |  |  | Reference |  |
| 2005-2009 | 0.99 (0.94-1.05) | 0.8 |  | 0.82 (0.81-0.83) | < 0.001 |
| 2010-2018 | 1.01 (0.96-1.07) | 0.7 |  | 0.69 (0.69-0.70) | < 0.001 |
| Number of primary cancers |  |  |  |  |  |
| Only one | Reference |  |  | Reference |  |
| MPCs | 1.07 (0.99-1.16) | 0.07 |  | 1.00 (0.99-1.01) | 0.5 |

Abbreviations: HR: Hazard ratios; API: Asian or Pacific Islander; AI/AN: American Indian/Alaska Native; MPCs: multiple primary cancers.

**Table S3** Suicides among patients with multiple primary cancers by combination of first primary cancer and second primary cancer.

| First primary cancer | Second primary cancer | No. of patients | Follow up in total (person-years) | Suicides | | | | |
| --- | --- | --- | --- | --- | --- | --- | --- | --- |
|  |  |  |  | Cancer population | | General population | | Smr (95% ci) |
|  |  |  |  | No. of observed deaths | Mortality rate (per 100,000 person-years) | No. of expected deaths | Mortality rate (per 100,000 person-years) |  |
| Oral cavity and pharynx | Lung and bronchus | 4,444 | 8,018 | 16 | 199.6 | 1.7 | 21.2 | 9.40 (5.76-15.3) |
| Prostate | Pancreas | 4,318 | 4,242 | 11 | 259.3 | 1.2 | 27.9 | 9.30 (5.15-16.8) |
| Oral cavity and pharynx | Oral cavity and pharynx | 5,230 | 17,045 | 20 | 117.3 | 3.5 | 20.8 | 5.64 (3.64-8.74) |
| Prostate | Esophagus | 1,939 | 3,298 | 5 | 151.6 | 1.0 | 28.8 | 5.26 (*2.19-12.6*) |
| Prostate | Lung and bronchus | 17,934 | 29,367 | 38 | 129.4 | 8.0 | 27.1 | 4.77 (3.47-6.56) |
| Lung and bronchus | Colon and rectum | 2,798 | 6,585 | 5 | 75.9 | 1.1 | 16.7 | 4.55 (1.89-10.9) |
| Prostate | Larynx | 1,263 | 5,182 | 6 | 115.8 | 1.4 | 26.4 | 4.39 (1.97-9.76) |
| Urinary bladder | Lung and bronchus | 6,903 | 11,236 | 11 | 97.9 | 2.7 | 23.9 | 4.10 (2.27-7.41) |
| Breast | Lung and bronchus | 12,846 | 30,354 | 6 | 19.8 | 1.7 | 5.7 | 3.44 (1.55-7.66) |
| Lung and bronchus | Prostate | 2,641 | 9,997 | 8 | 80.0 | 2.6 | 25.8 | 3.10 (1.55-6.20) |
| Prostate | Miscellaneous | 5,459 | 12,253 | 10 | 81.6 | 3.7 | 30.2 | 2.70 (1.45-5.02) |
| Non-basal skin | Non-Hodgkin lymphoma | 2,196 | 8,307 | 5 | 60.2 | 1.9 | 22.6 | 2.67 (1.11-6.41) |
| Colon and rectum | Non-basal skin | 1,885 | 8,360 | 5 | 59.8 | 1.9 | 23.2 | 2.57 (1.07-6.19) |
| Colon and rectum | Kidney and renal pelvis | 2,770 | 12,914 | 6 | 46.5 | 2.5 | 19.0 | 2.44 (1.10-5.44) |
| Colon and rectum | Lung and bronchus | 8,307 | 16,879 | 7 | 41.5 | 2.9 | 17.0 | 2.44 (1.16-5.11) |
| Urinary bladder | Colon and rectum | 2,341 | 8,165 | 5 | 61.2 | 2.2 | 26.5 | 2.31 (0.96-5.56) |
| Non-basal skin | Colon and rectum | 2,973 | 11,539 | 6 | 52.0 | 2.6 | 22.6 | 2.30 (1.03-5.13) |
| Urinary bladder | Prostate | 12,846 | 61,592 | 36 | 58.4 | 17.8 | 28.9 | 2.03 (1.46-2.81) |
| Colon and rectum | Colon and rectum | 22,020 | 93,754 | 32 | 34.1 | 16.1 | 17.2 | 1.99 (1.40-2.81) |
| Prostate | Colon and rectum | 12,297 | 49,782 | 25 | 50.2 | 13.6 | 27.3 | 1.84 (1.24-2.73) |
| Prostate | Urinary bladder | 13,784 | 61,100 | 30 | 49.1 | 18.3 | 30.0 | 1.64 (1.15-2.34) |
| Prostate | Oral cavity and pharynx | 3,115 | 11,403 | 5 | 43.8 | 3.2 | 28.0 | 1.56 (0.65-3.76) |
| Non-basal skin | Prostate | 8,342 | 45,703 | 21 | 45.9 | 13.6 | 29.7 | 1.54 (1.01-2.37) |
| Prostate | Non-basal skin | 7,790 | 36,369 | 16 | 44.0 | 11.5 | 31.6 | 1.39 (0.85-2.28) |
| Prostate | Non-Hodgkin lymphoma | 6,047 | 23,386 | 9 | 38.5 | 6.7 | 28.7 | 1.34 (0.70-2.58) |
| Breast | Breast | 58,831 | 310,834 | 26 | 8.4 | 20.1 | 6.5 | 1.30 (0.88-1.90) |
| Lung and bronchus | Lung and bronchus | 18,830 | 48,166 | 9 | 18.7 | 7.0 | 14.6 | 1.28 (0.67-2.46) |
| Colon and rectum | Prostate | 7,884 | 42,923 | 14 | 32.6 | 11.2 | 26.1 | 1.25 (0.74-2.11) |
| Non-basal skin | Non-basal skin | 19,859 | 98,824 | 24 | 24.3 | 22.3 | 22.6 | 1.07 (0.72-1.60) |
| Urinary bladder | Urinary bladder | 7,889 | 29,695 | 7 | 23.6 | 7.8 | 26.2 | 0.90 (0.43-1.89) |
| Prostate | Kidney and renal pelvis | 6,887 | 33,083 | 6 | 18.1 | 8.5 | 25.6 | 0.71 (0.32-1.58) |

**Table S4** Cardiovascular deaths among patients with multiple primary cancers by combination of first primary cancer and second primary cancer.

| First primary cancer | Second primary cancer | No. of patients | Follow up in total (person-years) | Suicides | | | | |
| --- | --- | --- | --- | --- | --- | --- | --- | --- |
|  |  |  |  | Cancer population | | General population | | SMR (95% ci) |
|  |  |  |  | No. of observed deaths | Mortality rate (per 100,000 person-years) | No. of expected deaths | Mortality rate (per 100,000 person-years) |  |
| Breast | Acute lymphocytic leukemia | 124 | 234 | 6 | 2,565.9 | 0.5 | 225.1 | 11.4 (5.12-25.4) |
| Brain | Brain | 518 | 1,431 | 8 | 558.9 | 0.9 | 65.9 | 8.48 (4.24-17.0) |
| Oral cavity and pharynx | Acute myeloid leukemia | 116 | 91 | 5 | 5,467.0 | 0.6 | 679.1 | 8.05 (3.35-19.3) |
| Oral cavity and pharynx | Chronic myeloid leukemia | 44 | 150 | 6 | 4,004.4 | 0.8 | 513.3 | 7.80 (3.51-17.4) |
| Anus | Vulva | 87 | 344 | 7 | 2,036.4 | 0.9 | 271.9 | 7.49 (3.57-15.7) |
| Kidney and renal pelvis | Acute myeloid leukemia | 128 | 172 | 8 | 4,664.7 | 1.1 | 644.2 | 7.24 (3.62-14.5) |
| Endocrine system | Hodgkin lymphoma | 45 | 262 | 5 | 1,909.6 | 0.7 | 266.5 | 7.17 (2.98-17.2) |
| Myeloma | Acute myeloid leukemia | 166 | 158 | 6 | 3,799.5 | 0.8 | 535.2 | 7.10 (3.19-15.8) |
| Esophagus | Larynx | 76 | 132 | 6 | 4,535.4 | 0.9 | 645.4 | 7.03 (3.16-15.6) |
| Small intestine | Myeloma | 27 | 78 | 5 | 6,420.5 | 0.7 | 958.3 | 6.70 (2.79-16.1) |
| Kidney and renal pelvis | Brain | 197 | 210 | 8 | 3,817.9 | 1.2 | 581.4 | 6.57 (3.28-13.1) |
| Larynx | Pancreas | 131 | 103 | 6 | 5,832.3 | 0.9 | 891.7 | 6.54 (2.94-14.6) |
| Hodgkin lymphoma | Oral cavity and pharynx | 77 | 262 | 6 | 2,287.2 | 0.9 | 351.2 | 6.51 (2.93-14.5) |
| Urinary bladder | Acute myeloid leukemia | 243 | 181 | 14 | 7,729.5 | 2.4 | 1,328.5 | 5.82 (3.45-9.82) |
| Ureter | Ureter | 39 | 148 | 5 | 3,383.1 | 0.9 | 586.0 | 5.77 (2.40-13.9) |
| Chronic myeloid leukemia | Acute myeloid leukemia | 395 | 324 | 9 | 2,778.8 | 1.6 | 489.0 | 5.68 (2.96-10.9) |
| Hodgkin lymphoma | Lung and bronchus | 267 | 469 | 16 | 3,409.4 | 2.9 | 611.9 | 5.57 (3.41-9.09) |
| Larynx | Liver | 155 | 173 | 6 | 3,459.9 | 1.1 | 654.3 | 5.29 (2.38-11.8) |
| Lung and bronchus | Brain | 199 | 174 | 6 | 3,451.6 | 1.2 | 685.9 | 5.03 (2.26-11.2) |
| Other biliary | Lung and bronchus | 134 | 177 | 8 | 4,526.2 | 1.6 | 899.5 | 5.03 (2.52-10.1) |
| Breast | Brain | 688 | 900 | 17 | 1,888.5 | 3.5 | 390.4 | 4.84 (3.01-7.78) |
| Colon and rectum | Other digestive organs | 97 | 120 | 6 | 5,008.7 | 1.3 | 1,045.3 | 4.79 (2.15-10.7) |
| Corpus uteri | Myeloma | 188 | 483 | 17 | 3,517.8 | 3.6 | 738.3 | 4.76 (2.96-7.66) |
| Breast | Acute myeloid leukemia | 1,249 | 2,123 | 29 | 1,365.9 | 6.1 | 286.7 | 4.76 (3.31-6.86) |
| Oral cavity and pharynx | Larynx | 553 | 1,593 | 45 | 2,824.6 | 9.5 | 596.2 | 4.74 (3.54-6.35) |
| Cervix uteri | Ovary | 145 | 581 | 5 | 860.6 | 1.1 | 183.9 | 4.68 (1.95-11.2) |
| Corpus uteri | Peritoneum | 114 | 396 | 5 | 1,263.3 | 1.1 | 273.5 | 4.62 (1.92-11.1) |
| Lung and bronchus | Acute myeloid leukemia | 374 | 262 | 7 | 2,675.6 | 1.6 | 595.2 | 4.50 (2.14-9.43) |
| Colon and rectum | Acute myeloid leukemia | 410 | 356 | 15 | 4,211.5 | 3.3 | 938.2 | 4.49 (2.71-7.45) |
| Acute myeloid leukemia | Urinary bladder | 50 | 158 | 6 | 3,792.5 | 1.3 | 849.5 | 4.46 (2.01-9.94) |
| Colon and rectum | Chronic myeloid leukemia | 199 | 538 | 30 | 5,577.9 | 6.8 | 1,255.5 | 4.44 (3.11-6.35) |
| Cervix uteri | Urinary bladder | 179 | 506 | 12 | 2,371.2 | 2.7 | 535.9 | 4.42 (2.51-7.79) |
| Vulva | Lung and bronchus | 764 | 1,405 | 33 | 2,348.3 | 7.5 | 533.4 | 4.40 (3.13-6.19) |
| Vulva | Ovary | 58 | 194 | 5 | 2,576.2 | 1.1 | 592.4 | 4.35 (1.81-10.4) |
| Colon and rectum | Brain | 375 | 450 | 14 | 3,112.0 | 3.3 | 729.2 | 4.27 (2.53-7.21) |
| Myeloma | Lung and bronchus | 656 | 865 | 35 | 4,045.3 | 8.2 | 948.5 | 4.27 (3.06-5.94) |
| Gallbladder | Urinary bladder | 23 | 68 | 6 | 8,812.7 | 1.4 | 2,086.9 | 4.22 (1.90-9.40) |
| Esophagus | Urinary bladder | 151 | 387 | 20 | 5,164.6 | 4.7 | 1,224.2 | 4.22 (2.72-6.54) |
| Oral cavity and pharynx | Esophagus | 673 | 953 | 28 | 2,937.4 | 6.7 | 701.3 | 4.19 (2.89-6.07) |
| Lung and bronchus | Chronic myeloid leukemia | 119 | 214 | 9 | 4,215.5 | 2.2 | 1,016.4 | 4.15 (2.16-7.97) |
| Kidney and renal pelvis | Esophagus | 178 | 286 | 12 | 4,197.6 | 3.0 | 1,038.5 | 4.04 (2.30-7.12) |
| Urinary bladder | Other leukemia | 58 | 163 | 8 | 4,900.5 | 2.0 | 1,246.7 | 3.93 (1.97-7.86) |
| Urinary bladder | Brain | 201 | 194 | 7 | 3,609.8 | 1.8 | 929.3 | 3.88 (1.85-8.15) |
| Vulva | Non-Hodgkin lymphoma | 125 | 456 | 9 | 1,972.4 | 2.3 | 510.4 | 3.86 (2.01-7.43) |
| Esophagus | Stomach | 317 | 565 | 19 | 3,365.8 | 5.0 | 878.6 | 3.83 (2.44-6.01) |
| Esophagus | Lung and bronchus | 647 | 1,018 | 34 | 3,340.3 | 9.0 | 883.8 | 3.78 (2.70-5.29) |
| Lung and bronchus | Larynx | 767 | 1,523 | 47 | 3,086.4 | 12.6 | 824.2 | 3.74 (2.81-4.98) |
| Chronic lymphocytic leukemia | Stomach | 102 | 126 | 6 | 4,746.2 | 1.6 | 1,276.1 | 3.72 (1.67-8.28) |
| Colon and rectum | Other leukemia | 108 | 202 | 10 | 4,939.3 | 2.7 | 1,329.6 | 3.71 (2.00-6.90) |
| Non-basal skin | Ureter | 43 | 124 | 5 | 4,028.2 | 1.3 | 1,084.8 | 3.71 (1.55-8.92) |
| Chronic myeloid leukemia | Non-basal skin | 81 | 293 | 8 | 2,729.6 | 2.2 | 747.7 | 3.65 (1.83-7.30) |
| Kidney and renal pelvis | Myeloma | 381 | 1,144 | 36 | 3,147.4 | 9.9 | 862.7 | 3.65 (2.63-5.06) |
| Cervix uteri | Lung and bronchus | 668 | 1,367 | 20 | 1,462.8 | 5.5 | 401.4 | 3.64 (2.35-5.65) |
| Non-Hodgkin lymphoma | Other biliary | 103 | 151 | 6 | 3,972.4 | 1.7 | 1,094.9 | 3.63 (1.63-8.08) |
| Larynx | Kidney and renal pelvis | 173 | 693 | 22 | 3,175.6 | 6.1 | 881.0 | 3.60 (2.37-5.47) |
| Larynx | Stomach | 97 | 139 | 6 | 4,314.0 | 1.7 | 1,197.3 | 3.60 (1.62-8.02) |
| Gallbladder | Breast | 69 | 201 | 8 | 3,987.5 | 2.2 | 1,116.8 | 3.57 (1.79-7.14) |
| Non-basal skin | Acute myeloid leukemia | 314 | 366 | 11 | 3,003.1 | 3.1 | 841.6 | 3.57 (1.98-6.44) |
| Lung and bronchus | Pancreas | 992 | 830 | 26 | 3,133.0 | 7.3 | 884.3 | 3.54 (2.41-5.20) |
| Pancreas | Pancreas | 197 | 373 | 8 | 2,147.7 | 2.3 | 613.9 | 3.50 (1.75-7.00) |
| Esophagus | Colon and rectum | 277 | 559 | 19 | 3,399.4 | 5.4 | 972.3 | 3.50 (2.23-5.48) |
| Anus | Lung and bronchus | 568 | 1,032 | 23 | 2,229.7 | 6.6 | 640.6 | 3.48 (2.31-5.24) |
| Non-Hodgkin lymphoma | Anus | 122 | 379 | 8 | 2,110.8 | 2.3 | 607.1 | 3.48 (1.74-6.95) |
| Chronic myeloid leukemia | Breast | 102 | 392 | 8 | 2,042.1 | 2.3 | 588.9 | 3.47 (1.73-6.93) |
| Breast | Vagina | 99 | 271 | 8 | 2,949.3 | 2.3 | 851.5 | 3.46 (1.73-6.93) |
| Corpus uteri | Stomach | 166 | 367 | 11 | 2,997.3 | 3.2 | 866.9 | 3.46 (1.91-6.24) |
| Vulva | Cervix uteri | 80 | 413 | 6 | 1,452.3 | 1.7 | 420.4 | 3.45 (1.55-7.69) |
| Cervix uteri | Non-Hodgkin lymphoma | 106 | 416 | 5 | 1,201.1 | 1.4 | 347.7 | 3.45 (1.44-8.30) |
| Lung and bronchus | Stomach | 624 | 736 | 30 | 4,078.6 | 8.8 | 1,199.0 | 3.40 (2.38-4.87) |
| Stomach | Small intestine | 130 | 387 | 11 | 2,839.0 | 3.3 | 854.1 | 3.32 (1.84-6.00) |
| Vulva | Non-basal skin | 117 | 518 | 10 | 1,929.0 | 3.1 | 589.7 | 3.27 (1.76-6.08) |
| Penis | Non-basal skin | 27 | 95 | 6 | 6,307.5 | 1.8 | 1,931.3 | 3.27 (1.47-7.27) |
| Eye and orbit | Chronic lymphocytic leukemia | 18 | 78 | 5 | 6,417.1 | 1.5 | 1,988.9 | 3.23 (1.34-7.75) |
| Urinary bladder | Liver | 568 | 763 | 25 | 3,277.6 | 7.9 | 1,032.2 | 3.18 (2.15-4.70) |
| Small intestine | Endocrine system | 78 | 354 | 6 | 1,694.1 | 1.9 | 536.1 | 3.16 (1.42-7.03) |
| Non-Hodgkin lymphoma | Chronic myeloid leukemia | 123 | 436 | 6 | 1,376.3 | 1.9 | 436.3 | 3.15 (1.42-7.02) |
| Lung and bronchus | Myeloma | 312 | 653 | 22 | 3,371.4 | 7.1 | 1,085.7 | 3.11 (2.04-4.72) |
| Corpus uteri | Pancreas | 459 | 471 | 10 | 2,122.6 | 3.2 | 685.0 | 3.10 (1.67-5.76) |
| Non-Hodgkin lymphoma | Acute myeloid leukemia | 788 | 1,050 | 18 | 1,714.0 | 5.8 | 553.6 | 3.10 (1.95-4.91) |
| Ureter | Breast | 44 | 200 | 6 | 2,998.8 | 1.9 | 974.5 | 3.08 (1.38-6.85) |
| Liver | Kidney and renal pelvis | 301 | 619 | 11 | 1,777.9 | 3.6 | 579.9 | 3.07 (1.70-5.54) |
| Chronic lymphocytic leukemia | Other leukemia | 81 | 224 | 10 | 4,464.3 | 3.3 | 1,459.7 | 3.06 (1.65-5.68) |
| Penis | Non-Hodgkin lymphoma | 39 | 120 | 6 | 4,994.8 | 2.0 | 1,636.2 | 3.05 (1.37-6.80) |
| Colon and rectum | Other biliary | 323 | 496 | 18 | 3,627.2 | 5.9 | 1,190.4 | 3.05 (1.92-4.84) |
| Oral cavity and pharynx | Lung and bronchus | 4,444 | 8,018 | 180 | 2,245.1 | 59.6 | 743.5 | 3.02 (2.61-3.49) |
| Chronic myeloid leukemia | Lung and bronchus | 208 | 231 | 7 | 3,036.3 | 2.3 | 1,006.4 | 3.02 (1.44-6.33) |
| Breast | Chronic myeloid leukemia | 350 | 1,327 | 26 | 1,958.9 | 8.7 | 653.2 | 3.00 (2.04-4.40) |
| Non-Hodgkin lymphoma | Stomach | 425 | 963 | 29 | 3,012.1 | 9.8 | 1,012.9 | 2.97 (2.07-4.28) |
| Penis | Lung and bronchus | 202 | 262 | 9 | 3,440.6 | 3.0 | 1,161.4 | 2.96 (1.54-5.69) |
| Kidney and renal pelvis | Other biliary | 94 | 137 | 5 | 3,644.1 | 1.7 | 1,247.3 | 2.92 (1.22-7.02) |
| Stomach | Myeloma | 57 | 140 | 7 | 5,006.0 | 2.4 | 1,716.7 | 2.92 (1.39-6.12) |
| Non-basal skin | Brain | 510 | 751 | 11 | 1,464.2 | 3.8 | 502.5 | 2.91 (1.61-5.26) |
| Nose, nasal cavity and middle ear | Oral cavity and pharynx | 80 | 229 | 5 | 2,181.4 | 1.7 | 749.1 | 2.91 (1.21-7.00) |
| Non-basal skin | Liver | 438 | 591 | 18 | 3,044.8 | 6.2 | 1,052.7 | 2.89 (1.82-4.59) |
| Chronic lymphocytic leukemia | Larynx | 41 | 140 | 6 | 4,276.8 | 2.1 | 1,483.5 | 2.88 (1.30-6.42) |
| Stomach | Corpus uteri | 55 | 176 | 6 | 3,401.0 | 2.1 | 1,180.8 | 2.88 (1.29-6.41) |
| Cervix uteri | Corpus uteri | 354 | 1,739 | 12 | 690.2 | 4.3 | 245.0 | 2.82 (1.60-4.96) |
| Chronic lymphocytic leukemia | Myeloma | 139 | 432 | 13 | 3,007.8 | 4.6 | 1,068.1 | 2.82 (1.64-4.85) |
| Vulva | Vagina | 91 | 342 | 7 | 2,045.3 | 2.5 | 729.6 | 2.80 (1.34-5.88) |
| Colon and rectum | Pancreas | 1,746 | 1,716 | 57 | 3,321.1 | 20.4 | 1,189.2 | 2.79 (2.15-3.62) |
| Myeloma | Colon and rectum | 416 | 937 | 31 | 3,308.6 | 11.1 | 1,185.1 | 2.79 (1.96-3.97) |
| Larynx | Endocrine system | 222 | 983 | 17 | 1,729.9 | 6.1 | 622.6 | 2.78 (1.73-4.47) |
| Small intestine | Lung and bronchus | 287 | 607 | 13 | 2,140.1 | 4.7 | 773.2 | 2.77 (1.61-4.77) |
| Urinary bladder | Small intestine | 169 | 463 | 14 | 3,026.2 | 5.1 | 1,094.0 | 2.77 (1.64-4.67) |
| Lung and bronchus | Esophagus | 600 | 647 | 20 | 3,091.4 | 7.3 | 1,122.1 | 2.75 (1.78-4.27) |
| Colon and rectum | Cervix uteri | 174 | 547 | 12 | 2,194.3 | 4.4 | 796.8 | 2.75 (1.56-4.85) |
| Lung and bronchus | Liver | 525 | 649 | 15 | 2,310.8 | 5.5 | 848.0 | 2.73 (1.64-4.52) |
| Kidney and renal pelvis | Larynx | 112 | 457 | 11 | 2,407.9 | 4.0 | 884.6 | 2.72 (1.51-4.92) |
| Penis | Penis | 162 | 636 | 19 | 2,989.6 | 7.0 | 1,103.1 | 2.71 (1.73-4.25) |
| Prostate | Brain | 1,362 | 1,366 | 35 | 2,562.5 | 13.0 | 952.5 | 2.69 (1.93-3.75) |
| Larynx | Non-Hodgkin lymphoma | 219 | 657 | 21 | 3,197.0 | 7.8 | 1,190.0 | 2.69 (1.75-4.12) |
| Chronic myeloid leukemia | Urinary bladder | 78 | 303 | 9 | 2,967.0 | 3.4 | 1,108.5 | 2.68 (1.39-5.14) |
| Liver | Lung and bronchus | 621 | 990 | 19 | 1,919.2 | 7.1 | 720.5 | 2.66 (1.70-4.18) |
| Cervix uteri | Colon and rectum | 303 | 1,000 | 9 | 900.4 | 3.4 | 338.1 | 2.66 (1.39-5.12) |
| Prostate | Acute myeloid leukemia | 1,228 | 1,516 | 44 | 2,903.3 | 16.6 | 1,093.8 | 2.65 (1.98-3.57) |
| Breast | Larynx | 224 | 844 | 18 | 2,132.1 | 6.8 | 803.9 | 2.65 (1.67-4.21) |
| Oral cavity and pharynx | Stomach | 236 | 422 | 11 | 2,606.1 | 4.2 | 985.8 | 2.64 (1.46-4.77) |
| Liver | Non-Hodgkin lymphoma | 252 | 537 | 6 | 1,117.4 | 2.3 | 423.5 | 2.64 (1.19-5.87) |
| Oral cavity and pharynx | Endocrine system | 572 | 2,897 | 25 | 863.0 | 9.5 | 329.5 | 2.62 (1.77-3.88) |
| Kidney and renal pelvis | Oral cavity and pharynx | 333 | 1,157 | 23 | 1,987.8 | 8.8 | 760.9 | 2.61 (1.74-3.93) |
| Hodgkin lymphoma | Breast | 157 | 672 | 6 | 892.6 | 2.3 | 342.1 | 2.61 (1.17-5.81) |
| Breast | Liver | 837 | 1,402 | 26 | 1,854.7 | 10.0 | 711.7 | 2.61 (1.77-3.83) |
| Colon and rectum | Hodgkin lymphoma | 83 | 329 | 8 | 2,429.8 | 3.1 | 932.6 | 2.61 (1.30-5.21) |
| Esophagus | Oral cavity and pharynx | 254 | 472 | 8 | 1,696.4 | 3.1 | 651.5 | 2.60 (1.30-5.21) |
| Endocrine system | Lung and bronchus | 1,264 | 3,220 | 51 | 1,583.8 | 19.6 | 609.9 | 2.60 (1.97-3.42) |
| Prostate | Other nervous system | 56 | 208 | 5 | 2,400.5 | 1.9 | 928.5 | 2.59 (1.08-6.21) |
| Pancreas | Non-basal skin | 88 | 237 | 5 | 2,113.8 | 1.9 | 818.1 | 2.58 (1.08-6.21) |
| Prostate | Other male genital organs | 88 | 440 | 15 | 3,405.5 | 5.8 | 1,318.7 | 2.58 (1.56-4.28) |
| Esophagus | Kidney and renal pelvis | 174 | 451 | 9 | 1,995.0 | 3.5 | 775.5 | 2.57 (1.34-4.94) |
| Liver | Urinary bladder | 150 | 335 | 8 | 2,389.2 | 3.1 | 932.1 | 2.56 (1.28-5.13) |
| Corpus uteri | Chronic lymphocytic leukemia | 123 | 559 | 13 | 2,326.8 | 5.1 | 912.9 | 2.55 (1.48-4.39) |
| Urinary bladder | Nose, nasal cavity and middle ear | 37 | 113 | 5 | 4,441.2 | 2.0 | 1,767.1 | 2.51 (1.05-6.04) |
| Larynx | Lung and bronchus | 3,189 | 5,483 | 113 | 2,060.9 | 45.0 | 821.2 | 2.51 (2.09-3.02) |
| Anus | Urinary bladder | 116 | 389 | 8 | 2,054.8 | 3.2 | 818.9 | 2.51 (1.25-5.02) |
| Stomach | Kidney and renal pelvis | 318 | 1,054 | 25 | 2,371.5 | 10.1 | 954.2 | 2.49 (1.68-3.68) |
| Non-basal skin | Larynx | 206 | 865 | 23 | 2,660.0 | 9.3 | 1,072.3 | 2.48 (1.65-3.73) |
| Kidney and renal pelvis | Pancreas | 623 | 801 | 17 | 2,121.1 | 6.9 | 856.2 | 2.48 (1.54-3.99) |
| Pancreas | Urinary bladder | 132 | 273 | 9 | 3,297.2 | 3.6 | 1,334.9 | 2.47 (1.29-4.75) |
| Corpus uteri | Ovary | 1,818 | 12,023 | 62 | 515.7 | 25.2 | 209.6 | 2.46 (1.92-3.16) |
| Breast | Ureter | 97 | 315 | 8 | 2,542.4 | 3.3 | 1,044.7 | 2.43 (1.22-4.87) |
| Urinary bladder | Lung and bronchus | 6,903 | 11,236 | 373 | 3,319.6 | 153.4 | 1,365.3 | 2.43 (2.20-2.69) |
| Nose, nasal cavity and middle ear | Nose, nasal cavity and middle ear | 119 | 360 | 6 | 1,666.3 | 2.5 | 689.0 | 2.42 (1.09-5.38) |
| Hodgkin lymphoma | Colon and rectum | 144 | 613 | 10 | 1,631.2 | 4.1 | 675.4 | 2.42 (1.30-4.49) |
| Oral cavity and pharynx | Myeloma | 109 | 326 | 7 | 2,147.2 | 2.9 | 892.0 | 2.41 (1.15-5.05) |
| Eye and orbit | Non-basal skin | 165 | 556 | 15 | 2,697.8 | 6.2 | 1,122.7 | 2.40 (1.45-3.99) |
| Larynx | Oral cavity and pharynx | 837 | 2,229 | 38 | 1,704.6 | 15.8 | 710.0 | 2.40 (1.75-3.30) |
| Hodgkin lymphoma | Urinary bladder | 71 | 272 | 5 | 1,840.5 | 2.1 | 769.2 | 2.39 (1.00-5.75) |
| Eye and orbit | Eye and orbit | 101 | 425 | 9 | 2,115.4 | 3.8 | 884.3 | 2.39 (1.24-4.60) |
| Lung and bronchus | Oral cavity and pharynx | 1,043 | 2,053 | 42 | 2,046.1 | 17.6 | 855.6 | 2.39 (1.77-3.24) |
| Chronic lymphocytic leukemia | Lung and bronchus | 1,282 | 1,910 | 53 | 2,775.5 | 22.2 | 1,163.2 | 2.39 (1.82-3.12) |
| Eye and orbit | Kidney and renal pelvis | 54 | 259 | 5 | 1,932.7 | 2.1 | 811.8 | 2.38 (0.99-5.72) |
| Kidney and renal pelvis | Kidney and renal pelvis | 5,195 | 24,298 | 337 | 1,387.0 | 142.4 | 586.0 | 2.37 (2.13-2.63) |
| Larynx | Breast | 154 | 585 | 10 | 1,710.4 | 4.2 | 724.2 | 2.36 (1.27-4.39) |
| Kidney and renal pelvis | Lung and bronchus | 2,683 | 5,820 | 128 | 2,199.2 | 54.3 | 933.0 | 2.36 (1.98-2.80) |
| Colon and rectum | Mesothelioma | 117 | 131 | 6 | 4,584.5 | 2.6 | 1,949.3 | 2.35 (1.06-5.24) |
| Prostate | Other digestive organs | 224 | 188 | 7 | 3,725.1 | 3.0 | 1,584.7 | 2.35 (1.12-4.93) |
| Pancreas | Lung and bronchus | 636 | 884 | 18 | 2,035.8 | 7.7 | 867.4 | 2.35 (1.48-3.73) |
| Nose, nasal cavity and middle ear | Lung and bronchus | 232 | 357 | 8 | 2,239.3 | 3.4 | 954.8 | 2.35 (1.17-4.69) |
| Non-basal skin | Stomach | 507 | 978 | 25 | 2,557.4 | 10.7 | 1,095.1 | 2.34 (1.58-3.46) |
| Myeloma | Kidney and renal pelvis | 262 | 682 | 12 | 1,760.5 | 5.1 | 753.9 | 2.34 (1.33-4.11) |
| Non-Hodgkin lymphoma | Liver | 438 | 791 | 11 | 1,390.4 | 4.7 | 598.3 | 2.32 (1.29-4.20) |
| Corpus uteri | Soft tissue including heart | 138 | 426 | 6 | 1,408.7 | 2.6 | 607.8 | 2.32 (1.04-5.16) |
| Vagina | Colon and rectum | 54 | 199 | 5 | 2,513.6 | 2.2 | 1,085.1 | 2.32 (0.96-5.57) |
| Kidney and renal pelvis | Liver | 506 | 958 | 14 | 1,460.7 | 6.1 | 635.8 | 2.30 (1.36-3.88) |
| Urinary bladder | Myeloma | 388 | 951 | 33 | 3,470.5 | 14.4 | 1,512.9 | 2.29 (1.63-3.23) |
| Stomach | Urinary bladder | 251 | 660 | 21 | 3,180.2 | 9.2 | 1,390.0 | 2.29 (1.49-3.51) |
| Oral cavity and pharynx | Oral cavity and pharynx | 5,230 | 17,045 | 260 | 1,525.4 | 113.9 | 668.2 | 2.28 (2.02-2.58) |
| Other urinary organs | Urinary bladder | 163 | 723 | 21 | 2,905.2 | 9.2 | 1,276.5 | 2.28 (1.48-3.49) |
| Anus | Kidney and renal pelvis | 104 | 440 | 6 | 1,363.4 | 2.6 | 599.6 | 2.27 (1.02-5.06) |
| Non-basal skin | Esophagus | 381 | 609 | 15 | 2,464.9 | 6.6 | 1,084.5 | 2.27 (1.37-3.77) |
| Lung and bronchus | Soft tissue including heart | 184 | 367 | 7 | 1,905.4 | 3.1 | 841.1 | 2.27 (1.08-4.75) |
| Prostate | Stomach | 2,605 | 5,505 | 178 | 3,233.4 | 78.7 | 1,429.1 | 2.26 (1.95-2.62) |
| Vulva | Corpus uteri | 156 | 826 | 7 | 847.9 | 3.1 | 375.1 | 2.26 (1.08-4.74) |
| Esophagus | Breast | 96 | 296 | 6 | 2,028.2 | 2.7 | 899.5 | 2.25 (1.01-5.02) |
| Soft tissue including heart | Kidney and renal pelvis | 184 | 751 | 11 | 1,464.2 | 4.9 | 651.5 | 2.25 (1.24-4.06) |
| Chronic myeloid leukemia | Colon and rectum | 127 | 453 | 9 | 1,987.7 | 4.0 | 888.4 | 2.24 (1.16-4.30) |
| Lung and bronchus | Colon and rectum | 2,798 | 6,585 | 180 | 2,733.4 | 80.6 | 1,223.4 | 2.23 (1.93-2.59) |
| Breast | Myeloma | 1,203 | 3,727 | 71 | 1,905.3 | 32.1 | 860.9 | 2.21 (1.75-2.79) |
| Breast | Pancreas | 2,973 | 2,973 | 53 | 1,782.9 | 24.0 | 806.4 | 2.21 (1.69-2.89) |
| Colon and rectum | Myeloma | 704 | 1,877 | 54 | 2,877.1 | 24.4 | 1,302.6 | 2.21 (1.69-2.88) |
| Lung and bronchus | Non-Hodgkin lymphoma | 1,248 | 3,221 | 75 | 2,328.3 | 34.0 | 1,055.2 | 2.21 (1.76-2.77) |
| Anus | Breast | 256 | 1,213 | 15 | 1,237.0 | 6.8 | 561.3 | 2.20 (1.33-3.66) |
| Non-Hodgkin lymphoma | Pancreas | 559 | 566 | 12 | 2,121.5 | 5.4 | 963.2 | 2.20 (1.25-3.88) |
| Stomach | Stomach | 855 | 2,529 | 42 | 1,660.5 | 19.1 | 754.7 | 2.20 (1.63-2.98) |
| Oral cavity and pharynx | Liver | 355 | 422 | 7 | 1,658.1 | 3.2 | 756.5 | 2.19 (1.04-4.60) |
| Kidney and renal pelvis | Endocrine system | 670 | 3,231 | 28 | 866.7 | 12.8 | 396.4 | 2.19 (1.51-3.17) |
| Corpus uteri | Kidney and renal pelvis | 659 | 2,882 | 31 | 1,075.8 | 14.3 | 494.9 | 2.17 (1.53-3.09) |
| Eye and orbit | Non-Hodgkin lymphoma | 67 | 216 | 7 | 3,247.6 | 3.2 | 1,495.4 | 2.17 (1.04-4.56) |
| Prostate | Penis | 188 | 596 | 23 | 3,858.5 | 10.6 | 1,780.7 | 2.17 (1.44-3.26) |
| Anus | Anus | 485 | 1,928 | 16 | 829.7 | 7.4 | 384.2 | 2.16 (1.32-3.53) |
| Breast | Esophagus | 418 | 700 | 14 | 1,999.4 | 6.5 | 932.5 | 2.14 (1.27-3.62) |
| Eye and orbit | Lung and bronchus | 189 | 375 | 8 | 2,132.6 | 3.8 | 1,000.5 | 2.13 (1.07-4.26) |
| Kidney and renal pelvis | Colon and rectum | 1,458 | 5,304 | 121 | 2,281.4 | 56.9 | 1,072.1 | 2.13 (1.78-2.54) |
| Larynx | Urinary bladder | 361 | 1,158 | 32 | 2,764.2 | 15.0 | 1,299.2 | 2.13 (1.50-3.01) |
| Breast | Hodgkin lymphoma | 162 | 716 | 12 | 1,676.6 | 5.6 | 788.1 | 2.13 (1.21-3.75) |
| Oral cavity and pharynx | Kidney and renal pelvis | 480 | 1,851 | 23 | 1,242.3 | 10.8 | 584.1 | 2.13 (1.41-3.20) |
| Prostate | Lung and bronchus | 17,934 | 29,367 | 854 | 2,908.0 | 402.2 | 1,369.6 | 2.12 (1.99-2.27) |
| Non-basal skin | Anus | 161 | 621 | 12 | 1,933.5 | 5.7 | 910.7 | 2.12 (1.21-3.74) |
| Colon and rectum | Esophagus | 614 | 1,073 | 26 | 2,422.7 | 12.3 | 1,143.9 | 2.12 (1.44-3.11) |
| Small intestine | Breast | 213 | 999 | 17 | 1,702.5 | 8.0 | 805.3 | 2.11 (1.31-3.40) |
| Larynx | Colon and rectum | 472 | 1,544 | 38 | 2,461.1 | 18.0 | 1,167.1 | 2.11 (1.53-2.90) |
| Small intestine | Non-basal skin | 83 | 359 | 8 | 2,228.9 | 3.8 | 1,059.0 | 2.10 (1.05-4.21) |
| Oral cavity and pharynx | Urinary bladder | 587 | 1,888 | 53 | 2,807.8 | 25.2 | 1,335.7 | 2.10 (1.61-2.75) |
| Ureter | Colon and rectum | 67 | 244 | 6 | 2,457.8 | 2.9 | 1,172.4 | 2.10 (0.94-4.67) |
| Urinary bladder | Pancreas | 862 | 759 | 22 | 2,898.9 | 10.5 | 1,384.1 | 2.09 (1.38-3.18) |
| Colon and rectum | Liver | 985 | 1,578 | 29 | 1,837.3 | 13.9 | 879.2 | 2.09 (1.45-3.01) |
| Kidney and renal pelvis | Stomach | 297 | 624 | 13 | 2,082.6 | 6.2 | 998.0 | 2.09 (1.21-3.59) |
| Non-Hodgkin lymphoma | Lung and bronchus | 3,426 | 7,123 | 146 | 2,049.8 | 70.2 | 985.3 | 2.08 (1.77-2.45) |
| Stomach | Esophagus | 221 | 382 | 8 | 2,096.1 | 3.8 | 1,007.7 | 2.08 (1.04-4.16) |
| Ovary | Kidney and renal pelvis | 179 | 693 | 8 | 1,154.4 | 3.9 | 559.0 | 2.07 (1.03-4.13) |
| Urinary bladder | Soft tissue including heart | 154 | 528 | 15 | 2,841.1 | 7.3 | 1,376.8 | 2.06 (1.24-3.42) |
| Gallbladder | Colon and rectum | 106 | 269 | 10 | 3,714.6 | 4.8 | 1,800.3 | 2.06 (1.11-3.83) |
| Chronic myeloid leukemia | Non-Hodgkin lymphoma | 99 | 237 | 5 | 2,109.0 | 2.4 | 1,022.4 | 2.06 (0.86-4.96) |
| Urinary bladder | Stomach | 514 | 912 | 29 | 3,179.5 | 14.1 | 1,550.3 | 2.05 (1.43-2.95) |
| Myeloma | Non-basal skin | 253 | 764 | 14 | 1,831.6 | 6.8 | 893.7 | 2.05 (1.21-3.46) |
| Corpus uteri | Colon and rectum | 1,839 | 7,483 | 110 | 1,469.9 | 53.7 | 718.1 | 2.05 (1.70-2.47) |
| Stomach | Lung and bronchus | 726 | 1,256 | 31 | 2,469.1 | 15.2 | 1,207.8 | 2.04 (1.44-2.91) |
| Liver | Colon and rectum | 233 | 439 | 6 | 1,368.2 | 2.9 | 669.4 | 2.04 (0.92-4.55) |
| Myeloma | Chronic lymphocytic leukemia | 150 | 500 | 11 | 2,199.1 | 5.4 | 1,076.1 | 2.04 (1.13-3.69) |
| Non-Hodgkin lymphoma | Myeloma | 419 | 1,155 | 22 | 1,905.4 | 10.8 | 933.2 | 2.04 (1.34-3.10) |
| Breast | Cervix uteri | 563 | 2,099 | 20 | 952.7 | 9.8 | 467.1 | 2.04 (1.32-3.16) |
| Colon and rectum | Gallbladder | 169 | 450 | 14 | 3,108.8 | 6.9 | 1,524.3 | 2.04 (1.21-3.44) |
| Breast | Lung and bronchus | 12,846 | 30,354 | 475 | 1,564.9 | 232.9 | 767.4 | 2.04 (1.86-2.23) |
| Corpus uteri | Endocrine system | 579 | 2,791 | 17 | 609.0 | 8.4 | 299.2 | 2.04 (1.27-3.27) |
| Cervix uteri | Breast | 651 | 3,115 | 21 | 674.2 | 10.3 | 331.5 | 2.03 (1.33-3.12) |
| Colon and rectum | Small intestine | 1,238 | 4,553 | 92 | 2,020.8 | 45.3 | 995.3 | 2.03 (1.66-2.49) |
| Corpus uteri | Lung and bronchus | 1,642 | 3,943 | 61 | 1,546.9 | 30.1 | 762.2 | 2.03 (1.58-2.61) |
| Urinary bladder | Larynx | 273 | 987 | 25 | 2,533.4 | 12.3 | 1,249.4 | 2.03 (1.37-3.00) |
| Lung and bronchus | Lung and bronchus | 18,830 | 48,166 | 817 | 1,696.2 | 404.7 | 840.2 | 2.02 (1.89-2.16) |
| Prostate | Liver | 2,115 | 3,181 | 74 | 2,326.3 | 36.7 | 1,152.4 | 2.02 (1.61-2.54) |
| Kidney and renal pelvis | Non-Hodgkin lymphoma | 819 | 3,155 | 61 | 1,933.2 | 30.3 | 959.5 | 2.01 (1.57-2.59) |
| Urinary bladder | Chronic myeloid leukemia | 108 | 256 | 9 | 3,521.4 | 4.5 | 1,748.9 | 2.01 (1.05-3.87) |
| Stomach | Non-basal skin | 135 | 392 | 11 | 2,804.3 | 5.5 | 1,397.4 | 2.01 (1.11-3.62) |
| Vagina | Vagina | 117 | 476 | 5 | 1,049.6 | 2.5 | 525.1 | 2.00 (0.83-4.80) |
| Prostate | Gallbladder | 232 | 362 | 12 | 3,313.8 | 6.0 | 1,665.3 | 1.99 (1.13-3.50) |
| Oral cavity and pharynx | Colon and rectum | 1,007 | 3,240 | 72 | 2,222.3 | 36.3 | 1,119.7 | 1.98 (1.58-2.50) |
| Prostate | Esophagus | 1,939 | 3,298 | 83 | 2,516.7 | 42.0 | 1,272.8 | 1.98 (1.59-2.45) |
| Myeloma | Urinary bladder | 227 | 643 | 18 | 2,798.7 | 9.1 | 1,417.4 | 1.97 (1.24-3.13) |
| Other leukemia | Colon and rectum | 77 | 278 | 7 | 2,520.3 | 3.5 | 1,277.5 | 1.97 (0.94-4.14) |
| Non-Hodgkin lymphoma | Hodgkin lymphoma | 399 | 1,613 | 12 | 744.2 | 6.1 | 378.1 | 1.97 (1.12-3.47) |
| Colon and rectum | Vulva | 121 | 484 | 14 | 2,894.6 | 7.1 | 1,473.3 | 1.96 (1.16-3.32) |
| Corpus uteri | Corpus uteri | 447 | 1,816 | 13 | 715.9 | 6.6 | 364.5 | 1.96 (1.14-3.38) |
| Prostate | Myeloma | 2,707 | 8,040 | 215 | 2,674.3 | 109.8 | 1,366.3 | 1.96 (1.71-2.24) |
| Non-Hodgkin lymphoma | Other leukemia | 182 | 600 | 8 | 1,332.5 | 4.1 | 684.8 | 1.95 (0.97-3.89) |
| Colon and rectum | Ovary | 520 | 1,541 | 23 | 1,492.4 | 11.8 | 768.8 | 1.94 (1.29-2.92) |
| Lung and bronchus | Urinary bladder | 2,060 | 5,253 | 143 | 2,722.2 | 73.9 | 1,406.4 | 1.94 (1.64-2.28) |
| Colon and rectum | Stomach | 1,206 | 2,734 | 71 | 2,596.5 | 36.7 | 1,343.2 | 1.93 (1.53-2.44) |
| Non-basal skin | Other biliary | 158 | 222 | 7 | 3,158.5 | 3.6 | 1,636.4 | 1.93 (0.92-4.05) |
| Urinary bladder | Other biliary | 155 | 213 | 6 | 2,818.0 | 3.1 | 1,462.8 | 1.93 (0.87-4.29) |
| Oral cavity and pharynx | Non-basal skin | 629 | 2,342 | 53 | 2,263.5 | 27.5 | 1,176.2 | 1.92 (1.47-2.52) |
| Myeloma | Non-Hodgkin lymphoma | 335 | 1,003 | 18 | 1,795.1 | 9.4 | 939.2 | 1.91 (1.20-3.03) |
| Pancreas | Colon and rectum | 295 | 482 | 9 | 1,866.4 | 4.7 | 977.3 | 1.91 (0.99-3.67) |
| Non-basal skin | Other leukemia | 103 | 405 | 7 | 1,730.4 | 3.7 | 910.7 | 1.90 (0.91-3.99) |
| Prostate | Chronic myeloid leukemia | 577 | 1,898 | 46 | 2,423.1 | 24.3 | 1,280.0 | 1.89 (1.42-2.53) |
| Non-basal skin | Myeloma | 643 | 1,947 | 43 | 2,208.1 | 22.8 | 1,169.3 | 1.89 (1.40-2.55) |
| Urinary bladder | Other urinary organs | 387 | 1,283 | 36 | 2,805.1 | 19.2 | 1,498.4 | 1.87 (1.35-2.60) |
| Non-basal skin | Lung and bronchus | 4,253 | 7,940 | 171 | 2,153.8 | 91.4 | 1,151.6 | 1.87 (1.61-2.17) |
| Lung and bronchus | Breast | 2,587 | 8,283 | 137 | 1,653.9 | 73.3 | 884.8 | 1.87 (1.58-2.21) |
| Lung and bronchus | Ovary | 257 | 563 | 7 | 1,243.1 | 3.7 | 665.3 | 1.87 (0.89-3.92) |
| Urinary bladder | Colon and rectum | 2,341 | 8,165 | 258 | 3,159.7 | 138.5 | 1,695.6 | 1.86 (1.65-2.11) |
| Lung and bronchus | Kidney and renal pelvis | 1,676 | 3,861 | 65 | 1,683.6 | 34.9 | 904.5 | 1.86 (1.46-2.37) |
| Pancreas | Kidney and renal pelvis | 305 | 470 | 6 | 1,275.9 | 3.2 | 688.2 | 1.85 (0.83-4.13) |
| Prostate | Other urinary organs | 223 | 502 | 16 | 3,185.4 | 8.6 | 1,720.2 | 1.85 (1.13-3.02) |
| Colon and rectum | Lung and bronchus | 8,307 | 16,879 | 374 | 2,215.8 | 203.8 | 1,207.7 | 1.83 (1.66-2.03) |
| Nose, nasal cavity and middle ear | Prostate | 86 | 386 | 7 | 1,811.3 | 3.8 | 987.4 | 1.83 (0.87-3.85) |
| Ovary | Corpus uteri | 1,947 | 11,845 | 39 | 329.3 | 21.3 | 180.0 | 1.83 (1.34-2.50) |
| Prostate | Pancreas | 4,318 | 4,242 | 97 | 2,286.7 | 53.1 | 1,251.1 | 1.83 (1.50-2.23) |
| Pancreas | Breast | 243 | 659 | 9 | 1,364.9 | 4.9 | 747.4 | 1.83 (0.95-3.51) |
| Stomach | Colon and rectum | 736 | 2,162 | 53 | 2,451.8 | 29.0 | 1,342.7 | 1.83 (1.39-2.39) |
| Chronic lymphocytic leukemia | Non-Hodgkin lymphoma | 1,023 | 2,887 | 51 | 1,766.5 | 28.0 | 968.7 | 1.82 (1.39-2.40) |
| Corpus uteri | Urinary bladder | 471 | 1,766 | 29 | 1,642.2 | 15.9 | 901.9 | 1.82 (1.27-2.62) |
| Breast | Other female genital organs | 403 | 1,238 | 12 | 968.9 | 6.6 | 532.7 | 1.82 (1.03-3.20) |
| Urinary bladder | Non-Hodgkin lymphoma | 1,089 | 3,679 | 95 | 2,582.1 | 52.5 | 1,426.0 | 1.81 (1.48-2.21) |
| Kidney and renal pelvis | Non-basal skin | 735 | 3,252 | 62 | 1,906.7 | 34.3 | 1,054.7 | 1.81 (1.41-2.32) |
| Endocrine system | Non-Hodgkin lymphoma | 532 | 2,329 | 19 | 815.8 | 10.5 | 452.0 | 1.80 (1.15-2.83) |
| Vulva | Vulva | 1,032 | 4,139 | 57 | 1,377.1 | 31.7 | 766.5 | 1.80 (1.39-2.33) |
| Colon and rectum | Colon and rectum | 22,020 | 93,754 | 1990 | 2,122.6 | 1112.6 | 1,186.7 | 1.79 (1.71-1.87) |
| Non-Hodgkin lymphoma | Oral cavity and pharynx | 573 | 1,816 | 30 | 1,651.8 | 16.9 | 928.2 | 1.78 (1.24-2.55) |
| Vulva | Urinary bladder | 107 | 439 | 6 | 1,368.2 | 3.4 | 770.5 | 1.78 (0.80-3.95) |
| Chronic lymphocytic leukemia | Oral cavity and pharynx | 188 | 442 | 10 | 2,264.8 | 5.7 | 1,279.8 | 1.77 (0.95-3.29) |
| Prostate | Other biliary | 611 | 948 | 26 | 2,741.5 | 14.7 | 1,551.3 | 1.77 (1.20-2.60) |
| Prostate | Other leukemia | 343 | 1,091 | 26 | 2,383.1 | 14.8 | 1,352.5 | 1.76 (1.20-2.59) |
| Small intestine | Colon and rectum | 414 | 1,494 | 24 | 1,606.6 | 13.6 | 913.1 | 1.76 (1.18-2.63) |
| Breast | Small intestine | 455 | 1,633 | 20 | 1,224.6 | 11.4 | 699.1 | 1.75 (1.13-2.72) |
| Lung and bronchus | Chronic lymphocytic leukemia | 314 | 721 | 17 | 2,357.3 | 9.8 | 1,352.2 | 1.74 (1.08-2.80) |
| Liver | Liver | 455 | 1,068 | 9 | 842.3 | 5.2 | 483.9 | 1.74 (0.91-3.35) |
| Colon and rectum | Larynx | 357 | 1,313 | 31 | 2,360.8 | 17.8 | 1,358.1 | 1.74 (1.22-2.47) |
| Lung and bronchus | Prostate | 2,641 | 9,997 | 195 | 1,950.6 | 112.4 | 1,124.3 | 1.73 (1.51-2.00) |
| Corpus uteri | Vagina | 238 | 759 | 8 | 1,053.5 | 4.6 | 608.3 | 1.73 (0.87-3.46) |
| Myeloma | Prostate | 717 | 2,717 | 52 | 1,913.8 | 30.2 | 1,109.8 | 1.72 (1.31-2.26) |
| Ovary | Lung and bronchus | 411 | 822 | 9 | 1,095.1 | 5.2 | 636.0 | 1.72 (0.90-3.31) |
| Kidney and renal pelvis | Corpus uteri | 323 | 1,259 | 11 | 873.6 | 6.4 | 508.0 | 1.72 (0.95-3.10) |
| Other leukemia | Breast | 59 | 294 | 6 | 2,043.1 | 3.5 | 1,190.1 | 1.72 (0.77-3.82) |
| Non-Hodgkin lymphoma | Esophagus | 216 | 355 | 8 | 2,255.6 | 4.7 | 1,316.5 | 1.71 (0.86-3.43) |
| Other leukemia | Non-Hodgkin lymphoma | 190 | 762 | 9 | 1,180.9 | 5.3 | 690.1 | 1.71 (0.89-3.29) |
| Larynx | Larynx | 604 | 2,447 | 37 | 1,512.1 | 21.6 | 884.5 | 1.71 (1.24-2.36) |
| Breast | Stomach | 1,244 | 2,820 | 40 | 1,418.7 | 23.4 | 831.3 | 1.71 (1.25-2.33) |
| Colon and rectum | Oral cavity and pharynx | 1,060 | 3,396 | 63 | 1,855.4 | 37.0 | 1,089.1 | 1.70 (1.33-2.18) |
| Non-Hodgkin lymphoma | Urinary bladder | 1,107 | 4,020 | 95 | 2,363.1 | 55.8 | 1,388.3 | 1.70 (1.39-2.08) |
| Prostate | Mesothelioma | 630 | 670 | 20 | 2,984.3 | 11.8 | 1,757.6 | 1.70 (1.10-2.63) |
| Colon and rectum | Kidney and renal pelvis | 2,770 | 12,914 | 215 | 1,664.8 | 126.8 | 981.8 | 1.70 (1.48-1.94) |
| Ovary | Non-Hodgkin lymphoma | 128 | 523 | 5 | 955.8 | 3.0 | 564.9 | 1.69 (0.70-4.07) |
| Prostate | Oral cavity and pharynx | 3,115 | 11,403 | 221 | 1,938.1 | 130.9 | 1,147.7 | 1.69 (1.48-1.93) |
| Urinary bladder | Urinary bladder | 7,889 | 29,695 | 748 | 2,519.0 | 443.2 | 1,492.4 | 1.69 (1.57-1.81) |
| Chronic myeloid leukemia | Prostate | 236 | 1,195 | 17 | 1,422.1 | 10.1 | 843.2 | 1.69 (1.05-2.71) |
| Colon and rectum | Non-basal skin | 1,885 | 8,360 | 202 | 2,416.1 | 119.9 | 1,433.7 | 1.69 (1.47-1.93) |
| Small intestine | Non-Hodgkin lymphoma | 87 | 334 | 5 | 1,496.6 | 3.0 | 893.6 | 1.67 (0.70-4.02) |
| Oral cavity and pharynx | Non-Hodgkin lymphoma | 557 | 1,953 | 30 | 1,536.3 | 17.9 | 919.1 | 1.67 (1.17-2.39) |
| Larynx | Non-basal skin | 170 | 708 | 14 | 1,978.3 | 8.4 | 1,184.3 | 1.67 (0.99-2.82) |
| Non-basal skin | Eye and orbit | 130 | 572 | 9 | 1,573.9 | 5.4 | 943.5 | 1.67 (0.87-3.21) |
| Non-Hodgkin lymphoma | Kidney and renal pelvis | 1,067 | 4,898 | 68 | 1,388.3 | 40.9 | 835.5 | 1.66 (1.31-2.11) |
| Urinary bladder | Kidney and renal pelvis | 2,211 | 8,821 | 173 | 1,961.2 | 104.5 | 1,185.1 | 1.65 (1.43-1.92) |
| Breast | Anus | 402 | 1,503 | 17 | 1,130.7 | 10.3 | 683.5 | 1.65 (1.03-2.66) |
| Breast | Soft tissue including heart | 726 | 2,593 | 29 | 1,118.3 | 17.5 | 676.1 | 1.65 (1.15-2.38) |
| Breast | Kidney and renal pelvis | 2,760 | 13,066 | 138 | 1,056.2 | 83.5 | 639.2 | 1.65 (1.40-1.95) |
| Non-Hodgkin lymphoma | Colon and rectum | 1,769 | 5,876 | 140 | 2,382.8 | 84.8 | 1,444.0 | 1.65 (1.40-1.95) |
| Breast | Oral cavity and pharynx | 1,394 | 5,355 | 62 | 1,157.7 | 37.6 | 701.8 | 1.65 (1.29-2.12) |
| Vulva | Breast | 741 | 3,837 | 34 | 886.1 | 20.6 | 538.1 | 1.65 (1.18-2.30) |
| Non-basal skin | Chronic myeloid leukemia | 150 | 452 | 10 | 2,211.0 | 6.1 | 1,344.6 | 1.64 (0.88-3.06) |
| Colon and rectum | Urinary bladder | 3,057 | 11,659 | 322 | 2,761.7 | 196.1 | 1,681.6 | 1.64 (1.47-1.83) |
| Larynx | Esophagus | 273 | 378 | 5 | 1,324.1 | 3.0 | 807.0 | 1.64 (0.68-3.94) |
| Oral cavity and pharynx | Nose, nasal cavity and middle ear | 120 | 373 | 5 | 1,341.1 | 3.1 | 818.7 | 1.64 (0.68-3.94) |
| Chronic lymphocytic leukemia | Corpus uteri | 121 | 421 | 7 | 1,661.4 | 4.3 | 1,020.8 | 1.63 (0.78-3.41) |
| Esophagus | Non-basal skin | 72 | 269 | 5 | 1,856.7 | 3.1 | 1,157.1 | 1.60 (0.67-3.86) |
| Kidney and renal pelvis | Urinary bladder | 2,896 | 14,778 | 275 | 1,860.9 | 171.5 | 1,160.3 | 1.60 (1.43-1.81) |
| Prostate | Bones and joints | 150 | 482 | 8 | 1,659.8 | 5.0 | 1,035.8 | 1.60 (0.80-3.20) |
| Penis | Colon and rectum | 63 | 198 | 5 | 2,525.8 | 3.1 | 1,586.9 | 1.59 (0.66-3.82) |
| Kidney and renal pelvis | Breast | 1,324 | 6,033 | 71 | 1,176.9 | 44.7 | 741.8 | 1.59 (1.26-2.00) |
| Myeloma | Myeloma | 827 | 2,652 | 21 | 791.7 | 13.3 | 500.1 | 1.58 (1.03-2.43) |
| Prostate | Breast | 328 | 1,452 | 31 | 2,134.9 | 19.6 | 1,348.6 | 1.58 (1.11-2.25) |
| Prostate | Nose, nasal cavity and middle ear | 192 | 626 | 11 | 1,756.3 | 7.0 | 1,111.6 | 1.58 (0.87-2.85) |
| Non-Hodgkin lymphoma | Small intestine | 122 | 421 | 6 | 1,423.6 | 3.8 | 905.0 | 1.57 (0.71-3.50) |
| Other biliary | Prostate | 97 | 386 | 7 | 1,813.3 | 4.5 | 1,154.7 | 1.57 (0.75-3.29) |
| Breast | Other biliary | 327 | 507 | 8 | 1,576.9 | 5.1 | 1,004.2 | 1.57 (0.79-3.14) |
| Colon and rectum | Chronic lymphocytic leukemia | 498 | 2,006 | 51 | 2,542.3 | 32.5 | 1,621.0 | 1.57 (1.19-2.06) |
| Non-basal skin | Mesothelioma | 132 | 169 | 5 | 2,961.5 | 3.2 | 1,888.9 | 1.57 (0.65-3.77) |
| Corpus uteri | Breast | 4,565 | 22,382 | 236 | 1,054.4 | 150.8 | 673.5 | 1.57 (1.38-1.78) |
| Prostate | Ureter | 182 | 511 | 13 | 2,542.8 | 8.3 | 1,624.7 | 1.57 (0.91-2.70) |
| Non-Hodgkin lymphoma | Non-Hodgkin lymphoma | 4,777 | 15,670 | 179 | 1,142.3 | 114.4 | 729.9 | 1.57 (1.35-1.81) |
| Stomach | Non-Hodgkin lymphoma | 228 | 611 | 13 | 2,127.2 | 8.3 | 1,360.9 | 1.56 (0.91-2.69) |
| Soft tissue including heart | Non-basal skin | 264 | 1,089 | 15 | 1,376.9 | 9.6 | 881.2 | 1.56 (0.94-2.59) |
| Anus | Colon and rectum | 324 | 1,183 | 14 | 1,183.8 | 9.0 | 757.7 | 1.56 (0.93-2.64) |
| Urinary bladder | Oral cavity and pharynx | 552 | 1,803 | 35 | 1,940.9 | 22.4 | 1,244.6 | 1.56 (1.12-2.17) |
| Colon and rectum | Corpus uteri | 1,527 | 6,586 | 73 | 1,108.4 | 46.8 | 711.0 | 1.56 (1.24-1.96) |
| Myeloma | Breast | 390 | 1,284 | 15 | 1,168.6 | 9.7 | 753.7 | 1.55 (0.93-2.57) |
| Other male genital organs | Prostate | 52 | 348 | 6 | 1,726.2 | 3.9 | 1,114.4 | 1.55 (0.70-3.45) |
| Lung and bronchus | Small intestine | 199 | 404 | 6 | 1,483.8 | 3.9 | 959.4 | 1.55 (0.69-3.44) |
| Endocrine system | Endocrine system | 1,138 | 6,993 | 29 | 414.7 | 18.8 | 268.2 | 1.55 (1.07-2.23) |
| Larynx | Prostate | 870 | 4,294 | 75 | 1,746.7 | 48.9 | 1,139.2 | 1.53 (1.22-1.92) |
| Breast | Non-Hodgkin lymphoma | 3,725 | 15,902 | 211 | 1,326.9 | 137.7 | 865.7 | 1.53 (1.34-1.75) |
| Non-basal skin | Oral cavity and pharynx | 1,057 | 4,048 | 62 | 1,531.5 | 40.5 | 1,001.3 | 1.53 (1.19-1.96) |
| Chronic lymphocytic leukemia | Kidney and renal pelvis | 353 | 1,383 | 21 | 1,518.9 | 13.7 | 993.3 | 1.53 (1.00-2.35) |
| Non-basal skin | Pancreas | 1,158 | 1,136 | 18 | 1,584.7 | 11.8 | 1,043.2 | 1.52 (0.96-2.41) |
| Corpus uteri | Cervix uteri | 281 | 1,435 | 9 | 627.4 | 5.9 | 413.5 | 1.52 (0.79-2.92) |
| Urinary bladder | Prostate | 12,846 | 61,592 | 1116 | 1,811.9 | 737.7 | 1,197.8 | 1.51 (1.43-1.60) |
| Stomach | Breast | 323 | 1,153 | 19 | 1,648.5 | 12.6 | 1,097.5 | 1.50 (0.96-2.35) |
| Prostate | Larynx | 1,263 | 5,182 | 108 | 2,084.3 | 72.0 | 1,388.7 | 1.50 (1.24-1.81) |
| Colon and rectum | Non-Hodgkin lymphoma | 2,237 | 8,375 | 179 | 2,137.3 | 119.4 | 1,425.8 | 1.50 (1.29-1.74) |
| Non-Hodgkin lymphoma | Corpus uteri | 382 | 1,656 | 17 | 1,026.9 | 11.4 | 685.7 | 1.50 (0.93-2.41) |
| Ureter | Kidney and renal pelvis | 136 | 595 | 10 | 1,679.4 | 6.7 | 1,129.9 | 1.49 (0.80-2.76) |
| Breast | Vulva | 430 | 1,670 | 26 | 1,556.7 | 17.5 | 1,047.4 | 1.49 (1.01-2.18) |
| Endocrine system | Kidney and renal pelvis | 659 | 2,801 | 17 | 606.9 | 11.5 | 409.3 | 1.48 (0.92-2.39) |
| Other leukemia | Prostate | 181 | 997 | 14 | 1,404.8 | 9.5 | 949.6 | 1.48 (0.88-2.50) |
| Breast | Chronic lymphocytic leukemia | 796 | 3,744 | 60 | 1,602.7 | 40.6 | 1,083.5 | 1.48 (1.15-1.91) |
| Colon and rectum | Soft tissue including heart | 308 | 1,028 | 19 | 1,847.4 | 12.9 | 1,249.8 | 1.48 (0.94-2.32) |
| Lung and bronchus | Endocrine system | 808 | 3,113 | 32 | 1,027.9 | 21.7 | 698.5 | 1.47 (1.04-2.08) |
| Penis | Prostate | 187 | 1,003 | 17 | 1,694.4 | 11.6 | 1,155.4 | 1.47 (0.91-2.36) |
| Breast | Corpus uteri | 6,348 | 30,120 | 254 | 843.3 | 173.4 | 575.8 | 1.46 (1.30-1.66) |
| Non-basal skin | Kidney and renal pelvis | 1,468 | 6,236 | 81 | 1,299.0 | 55.4 | 888.1 | 1.46 (1.18-1.82) |
| Small intestine | Prostate | 344 | 1,760 | 25 | 1,420.6 | 17.1 | 972.9 | 1.46 (0.99-2.16) |
| Lung and bronchus | Non-basal skin | 773 | 2,471 | 42 | 1,699.9 | 28.8 | 1,165.6 | 1.46 (1.08-1.97) |
| Kidney and renal pelvis | Chronic lymphocytic leukemia | 235 | 956 | 15 | 1,569.7 | 10.3 | 1,080.0 | 1.45 (0.88-2.41) |
| Breast | Urinary bladder | 2,119 | 8,831 | 144 | 1,630.6 | 99.1 | 1,122.5 | 1.45 (1.23-1.71) |
| Soft tissue including heart | Lung and bronchus | 399 | 992 | 12 | 1,209.4 | 8.3 | 834.7 | 1.45 (0.82-2.55) |
| Esophagus | Esophagus | 293 | 636 | 13 | 2,043.4 | 9.0 | 1,414.5 | 1.44 (0.84-2.49) |
| Soft tissue including heart | Urinary bladder | 117 | 467 | 9 | 1,926.7 | 6.2 | 1,335.9 | 1.44 (0.75-2.77) |
| Esophagus | Prostate | 382 | 1,556 | 25 | 1,606.8 | 17.3 | 1,114.6 | 1.44 (0.97-2.13) |
| Breast | Colon and rectum | 7,846 | 32,758 | 501 | 1,529.4 | 347.9 | 1,062.0 | 1.44 (1.32-1.57) |
| Urinary bladder | Corpus uteri | 245 | 988 | 11 | 1,113.6 | 7.7 | 776.0 | 1.44 (0.79-2.59) |
| Oral cavity and pharynx | Prostate | 1,984 | 9,538 | 109 | 1,142.8 | 76.1 | 798.3 | 1.43 (1.19-1.73) |
| Soft tissue including heart | Colon and rectum | 189 | 688 | 12 | 1,743.8 | 8.4 | 1,222.7 | 1.43 (0.81-2.51) |
| Eye and orbit | Colon and rectum | 80 | 267 | 6 | 2,246.5 | 4.2 | 1,576.4 | 1.43 (0.64-3.17) |
| Urinary bladder | Non-basal skin | 1,124 | 4,470 | 114 | 2,550.5 | 80.1 | 1,792.6 | 1.42 (1.18-1.71) |
| Vulva | Colon and rectum | 222 | 785 | 10 | 1,274.0 | 7.0 | 896.8 | 1.42 (0.76-2.64) |
| Non-basal skin | Vulva | 94 | 432 | 6 | 1,387.8 | 4.2 | 978.0 | 1.42 (0.64-3.16) |
| Corpus uteri | Vulva | 112 | 438 | 6 | 1,369.2 | 4.2 | 966.3 | 1.42 (0.64-3.15) |
| Breast | Gallbladder | 259 | 462 | 7 | 1,515.3 | 4.9 | 1,070.4 | 1.42 (0.67-2.97) |
| Pancreas | Prostate | 288 | 905 | 14 | 1,547.5 | 9.9 | 1,093.3 | 1.42 (0.84-2.39) |
| Prostate | Hodgkin lymphoma | 246 | 1,070 | 14 | 1,308.2 | 9.9 | 926.0 | 1.41 (0.84-2.39) |
| Prostate | Urinary bladder | 13,784 | 61,100 | 1340 | 2,193.1 | 953.2 | 1,560.1 | 1.41 (1.33-1.48) |
| Ureter | Urinary bladder | 852 | 4,467 | 84 | 1,880.4 | 59.8 | 1,339.4 | 1.40 (1.13-1.74) |
| Oral cavity and pharynx | Chronic lymphocytic leukemia | 104 | 407 | 6 | 1,475.1 | 4.3 | 1,051.1 | 1.40 (0.63-3.12) |
| Non-Hodgkin lymphoma | Soft tissue including heart | 137 | 431 | 6 | 1,393.7 | 4.3 | 997.0 | 1.40 (0.63-3.11) |
| Prostate | Colon and rectum | 12,297 | 49,782 | 1044 | 2,097.1 | 750.1 | 1,506.8 | 1.39 (1.31-1.48) |
| Breast | Breast | 58,831 | 310,834 | 2474 | 795.9 | 1780.9 | 572.9 | 1.39 (1.34-1.45) |
| Lung and bronchus | Corpus uteri | 347 | 910 | 9 | 989.3 | 6.5 | 714.9 | 1.38 (0.72-2.66) |
| Corpus uteri | Oral cavity and pharynx | 151 | 464 | 6 | 1,293.7 | 4.3 | 936.9 | 1.38 (0.62-3.07) |
| Non-basal skin | Colon and rectum | 2,973 | 11,539 | 224 | 1,941.2 | 163.3 | 1,414.8 | 1.37 (1.20-1.56) |
| Colon and rectum | Breast | 5,039 | 24,261 | 380 | 1,566.3 | 277.5 | 1,143.9 | 1.37 (1.24-1.51) |
| Urinary bladder | Esophagus | 382 | 560 | 11 | 1,965.5 | 8.1 | 1,439.5 | 1.37 (0.76-2.47) |
| Vagina | Breast | 124 | 612 | 5 | 817.4 | 3.7 | 598.7 | 1.37 (0.57-3.28) |
| Non-Hodgkin lymphoma | Non-basal skin | 1,222 | 4,802 | 78 | 1,624.4 | 57.2 | 1,190.3 | 1.36 (1.09-1.70) |
| Prostate | Non-Hodgkin lymphoma | 6,047 | 23,386 | 413 | 1,766.0 | 303.4 | 1,297.4 | 1.36 (1.24-1.50) |
| Urinary bladder | Ureter | 779 | 3,166 | 56 | 1,768.5 | 41.2 | 1,300.0 | 1.36 (1.05-1.77) |
| Prostate | Chronic lymphocytic leukemia | 2,010 | 8,799 | 172 | 1,954.8 | 127.1 | 1,444.9 | 1.35 (1.17-1.57) |
| Hodgkin lymphoma | Non-Hodgkin lymphoma | 484 | 2,021 | 10 | 494.9 | 7.4 | 366.3 | 1.35 (0.73-2.51) |
| Prostate | Kidney and renal pelvis | 6,887 | 33,083 | 462 | 1,396.5 | 343.3 | 1,037.8 | 1.35 (1.23-1.47) |
| Prostate | Small intestine | 769 | 2,738 | 43 | 1,570.5 | 32.0 | 1,167.4 | 1.35 (1.00-1.81) |
| Non-basal skin | Non-Hodgkin lymphoma | 2,196 | 8,307 | 129 | 1,553.0 | 96.2 | 1,157.6 | 1.34 (1.13-1.59) |
| Urinary bladder | Breast | 1,134 | 5,390 | 85 | 1,577.1 | 63.4 | 1,176.4 | 1.34 (1.08-1.66) |
| Ovary | Breast | 1,163 | 5,020 | 36 | 717.2 | 26.9 | 535.0 | 1.34 (0.97-1.86) |
| Non-Hodgkin lymphoma | Breast | 1,789 | 8,202 | 104 | 1,268.0 | 77.8 | 948.0 | 1.34 (1.10-1.62) |
| Other urinary organs | Prostate | 164 | 624 | 10 | 1,601.5 | 7.5 | 1,202.3 | 1.33 (0.72-2.48) |
| Non-basal skin | Ovary | 351 | 1,136 | 7 | 616.2 | 5.3 | 462.8 | 1.33 (0.63-2.79) |
| Non-basal skin | Chronic lymphocytic leukemia | 793 | 3,543 | 61 | 1,721.7 | 46.1 | 1,302.0 | 1.32 (1.03-1.70) |
| Prostate | Anus | 300 | 997 | 18 | 1,805.7 | 13.6 | 1,367.1 | 1.32 (0.83-2.10) |
| Soft tissue including heart | Soft tissue including heart | 496 | 1,779 | 13 | 730.9 | 9.9 | 557.3 | 1.31 (0.76-2.26) |
| Non-basal skin | Non-basal skin | 19,859 | 98,824 | 1187 | 1,201.1 | 909.9 | 920.7 | 1.30 (1.23-1.38) |
| Chronic lymphocytic leukemia | Non-basal skin | 707 | 2,542 | 43 | 1,691.7 | 33.3 | 1,310.2 | 1.29 (0.96-1.74) |
| Urinary bladder | Endocrine system | 271 | 1,361 | 14 | 1,028.5 | 10.9 | 799.9 | 1.29 (0.76-2.17) |
| Small intestine | Small intestine | 174 | 640 | 6 | 938.0 | 4.7 | 729.5 | 1.29 (0.58-2.86) |
| Soft tissue including heart | Breast | 252 | 1,234 | 12 | 972.4 | 9.4 | 760.4 | 1.28 (0.73-2.25) |
| Urinary bladder | Chronic lymphocytic leukemia | 343 | 1,370 | 33 | 2,408.4 | 25.8 | 1,885.5 | 1.28 (0.91-1.80) |
| Non-Hodgkin lymphoma | Endocrine system | 827 | 4,513 | 25 | 553.9 | 19.7 | 435.8 | 1.27 (0.86-1.88) |
| Eye and orbit | Prostate | 167 | 871 | 12 | 1,377.3 | 9.5 | 1,088.8 | 1.27 (0.72-2.23) |
| Non-basal skin | Urinary bladder | 2,180 | 8,730 | 181 | 2,073.4 | 144.3 | 1,652.7 | 1.25 (1.08-1.45) |
| Corpus uteri | Non-Hodgkin lymphoma | 658 | 2,890 | 23 | 795.9 | 18.4 | 635.8 | 1.25 (0.83-1.88) |
| Soft tissue including heart | Non-Hodgkin lymphoma | 140 | 425 | 6 | 1,412.9 | 4.8 | 1,130.3 | 1.25 (0.56-2.78) |
| Oral cavity and pharynx | Breast | 691 | 3,018 | 28 | 927.6 | 22.5 | 745.5 | 1.24 (0.86-1.80) |
| Ovary | Colon and rectum | 536 | 1,877 | 15 | 799.1 | 12.1 | 643.8 | 1.24 (0.75-2.06) |
| Endocrine system | Non-basal skin | 635 | 2,989 | 16 | 535.3 | 12.9 | 432.8 | 1.24 (0.76-2.02) |
| Non-basal skin | Corpus uteri | 819 | 3,757 | 27 | 718.7 | 22.0 | 585.4 | 1.23 (0.84-1.79) |
| Kidney and renal pelvis | Prostate | 3,770 | 20,160 | 230 | 1,140.9 | 188.2 | 933.4 | 1.22 (1.07-1.39) |
| Colon and rectum | Prostate | 7,884 | 42,923 | 638 | 1,486.4 | 525.6 | 1,224.5 | 1.21 (1.12-1.31) |
| Testis | Prostate | 229 | 1,234 | 5 | 405.2 | 4.1 | 335.2 | 1.21 (0.50-2.90) |
| Chronic lymphocytic leukemia | Urinary bladder | 493 | 1,689 | 33 | 1,953.8 | 27.4 | 1,620.3 | 1.21 (0.86-1.70) |
| Prostate | Non-basal skin | 7,790 | 36,369 | 592 | 1,627.8 | 498.8 | 1,371.5 | 1.19 (1.10-1.29) |
| Breast | Endocrine system | 3,384 | 18,547 | 65 | 350.5 | 54.9 | 296.2 | 1.18 (0.93-1.51) |
| Colon and rectum | Endocrine system | 1,055 | 5,365 | 34 | 633.7 | 28.9 | 537.7 | 1.18 (0.84-1.65) |
| Non-basal skin | Small intestine | 182 | 642 | 7 | 1,090.6 | 6.0 | 927.7 | 1.18 (0.56-2.47) |
| Non-Hodgkin lymphoma | Chronic lymphocytic leukemia | 573 | 2,525 | 30 | 1,187.9 | 25.6 | 1,013.9 | 1.17 (0.82-1.68) |
| Chronic lymphocytic leukemia | Prostate | 1,233 | 6,265 | 82 | 1,308.8 | 70.3 | 1,122.6 | 1.17 (0.94-1.45) |
| Corpus uteri | Non-basal skin | 521 | 2,391 | 21 | 878.2 | 18.1 | 757.8 | 1.16 (0.76-1.78) |
| Non-Hodgkin lymphoma | Prostate | 2,713 | 13,579 | 163 | 1,200.4 | 141.8 | 1,044.4 | 1.15 (0.99-1.34) |
| Non-basal skin | Endocrine system | 1,258 | 6,752 | 31 | 459.1 | 27.0 | 400.3 | 1.15 (0.81-1.63) |
| Colon and rectum | Anus | 491 | 1,966 | 18 | 915.7 | 15.7 | 798.5 | 1.15 (0.72-1.82) |
| Prostate | Soft tissue including heart | 940 | 3,269 | 55 | 1,682.3 | 48.1 | 1,471.2 | 1.14 (0.88-1.49) |
| Endocrine system | Urinary bladder | 318 | 1,402 | 15 | 1,069.7 | 13.2 | 939.9 | 1.14 (0.69-1.89) |
| Endocrine system | Colon and rectum | 785 | 3,548 | 26 | 732.8 | 22.9 | 644.2 | 1.14 (0.77-1.67) |
| Small intestine | Urinary bladder | 111 | 451 | 7 | 1,552.2 | 6.2 | 1,375.0 | 1.13 (0.54-2.37) |
| Breast | Ovary | 2,618 | 9,507 | 51 | 536.4 | 45.3 | 476.3 | 1.13 (0.86-1.48) |
| Ureter | Prostate | 122 | 560 | 8 | 1,428.7 | 7.1 | 1,270.4 | 1.12 (0.56-2.25) |
| Prostate | Eye and orbit | 184 | 842 | 11 | 1,305.8 | 9.9 | 1,180.1 | 1.11 (0.61-2.00) |
| Breast | Non-basal skin | 3,757 | 18,909 | 160 | 846.1 | 144.6 | 765.0 | 1.11 (0.95-1.29) |
| Ureter | Lung and bronchus | 178 | 386 | 5 | 1,294.1 | 4.5 | 1,176.7 | 1.10 (0.46-2.64) |
| Non-Hodgkin lymphoma | Larynx | 149 | 481 | 5 | 1,040.3 | 4.6 | 959.1 | 1.08 (0.45-2.61) |
| Prostate | Endocrine system | 1,373 | 6,737 | 67 | 994.5 | 61.9 | 918.5 | 1.08 (0.85-1.38) |
| Endocrine system | Corpus uteri | 520 | 2,230 | 7 | 314.0 | 6.5 | 292.1 | 1.07 (0.51-2.25) |
| Soft tissue including heart | Prostate | 378 | 1,982 | 24 | 1,210.7 | 23.1 | 1,165.9 | 1.04 (0.70-1.55) |
| Endocrine system | Breast | 2,393 | 11,695 | 46 | 393.3 | 44.4 | 379.9 | 1.04 (0.78-1.38) |
| Breast | Prostate | 278 | 1,445 | 15 | 1,037.9 | 14.8 | 1,020.8 | 1.02 (0.61-1.69) |
| Prostate | Kaposi sarcoma | 75 | 375 | 7 | 1,868.7 | 7.0 | 1,879.6 | 0.99 (0.47-2.09) |
| Non-basal skin | Breast | 4,476 | 22,875 | 146 | 638.3 | 150.1 | 656.1 | 0.97 (0.83-1.14) |
| Non-basal skin | Soft tissue including heart | 454 | 1,582 | 17 | 1,074.6 | 17.5 | 1,106.7 | 0.97 (0.60-1.56) |
| Chronic lymphocytic leukemia | Breast | 548 | 2,391 | 25 | 1,045.6 | 26.4 | 1,103.0 | 0.95 (0.64-1.40) |
| Chronic lymphocytic leukemia | Colon and rectum | 608 | 1,905 | 29 | 1,522.3 | 30.7 | 1,610.1 | 0.95 (0.66-1.36) |
| Non-basal skin | Prostate | 8,342 | 45,703 | 440 | 962.7 | 465.5 | 1,018.5 | 0.95 (0.86-1.04) |
| Endocrine system | Prostate | 1,069 | 5,694 | 38 | 667.4 | 41.3 | 725.9 | 0.92 (0.67-1.26) |
| Liver | Prostate | 346 | 1,105 | 7 | 633.7 | 8.1 | 733.9 | 0.86 (0.41-1.81) |
| Stomach | Prostate | 592 | 2,572 | 26 | 1,011.1 | 30.2 | 1,173.0 | 0.86 (0.59-1.27) |
| Anus | Prostate | 175 | 788 | 6 | 761.2 | 7.0 | 886.0 | 0.86 (0.39-1.91) |
| Breast | Eye and orbit | 161 | 807 | 5 | 619.7 | 5.9 | 731.9 | 0.85 (0.35-2.03) |
| Hodgkin lymphoma | Prostate | 179 | 965 | 6 | 621.5 | 7.2 | 748.6 | 0.83 (0.37-1.85) |
